# Current State and Promising Opportunities on Pharmaceutical Approaches in the Treatment of Polymicrobial Diseases

**DOI:** 10.3390/pathogens10020245

**Published:** 2021-02-20

**Authors:** Sartini Sartini, Andi Dian Permana, Saikat Mitra, Abu Montakim Tareq, Emil Salim, Islamudin Ahmad, Harapan Harapan, Talha Bin Emran, Firzan Nainu

**Affiliations:** 1 Faculty of Pharmacy, Hasanuddin University, Makassar 90245, Indonesia; sardj@farmasi.unhas.ac.id (S.S.); andi.dian.permana@farmasi.unhas.ac.id (A.D.P.); 2 Department of Pharmacy, Faculty of Pharmacy, University of Dhaka, Dhaka 1000, Bangladesh; saikatmitradu@gmail.com or; 3 Department of Pharmacy, International Islamic University Chittagong, Chittagong 4318, Bangladesh; montakim0.abu@gmail.com or; 4 Faculty of Pharmacy, Universitas Sumatera Utara, North Sumatera 20155, Indonesia; emilsalim@usu.ac.id; 5 Faculty of Pharmacy, Universitas Mulawarman, East Kalimantan 75119, Indonesia; islamudinahmad@farmasi.unmul.ac.id; 6 Medical Research Unit, School of Medicine, Universitas Syiah Kuala, Banda Aceh 23111, Indonesia; harapan@unsyiah.ac.id; 7 Tropical Disease Centre, School of Medicine, Universitas Syiah Kuala, Banda Aceh 23111, Indonesia; 8 Department of Microbiology, School of Medicine, Universitas Syiah Kuala, Banda Aceh 23111, Indonesia; 9 Department of Pharmacy, BGC Trust University Bangladesh, Chittagong 4381, Bangladesh; talhabmb@bgctub.ac.bd

**Keywords:** polymicrobial diseases, biofilms, antimicrobials, natural products, pharmacological approach, drug delivery system

## Abstract

In recent years, the emergence of newly identified acute and chronic infectious disorders caused by diverse combinations of pathogens, termed polymicrobial diseases, has had catastrophic consequences for humans. Antimicrobial agents have been clinically proven to be effective in the pharmacological treatment of polymicrobial diseases. Unfortunately, an increasing trend in the emergence of multi-drug-resistant pathogens and limited options for delivery of antimicrobial drugs might seriously impact humans’ efforts to combat polymicrobial diseases in the coming decades. New antimicrobial agents with novel mechanism(s) of action and new pharmaceutical formulations or delivery systems to target infected sites are urgently required. In this review, we discuss the prospective use of novel antimicrobial compounds isolated from natural products to treat polymicrobial infections, mainly via mechanisms related to inhibition of biofilm formation. Drug-delivery systems developed to deliver antimicrobial compounds to both intracellular and extracellular pathogens are discussed. We further discuss the effectiveness of several biofilm-targeted delivery strategies to eliminate polymicrobial biofilms. At the end, we review the applications and promising opportunities for various drug-delivery systems, when compared to conventional antimicrobial therapy, as a pharmacological means to treat polymicrobial diseases.

## 1. Introduction

Microorganisms commonly grow in multifaceted polymicrobial biofilm communities in nature, attached to host mucosal sites and environmental surfaces [1]. By definition, polymicrobial biofilm communities comprise multiple microbial organisms (fungi, bacteria, and viruses) inhabiting a matrix that consists of microbes’ metabolic products and/or host-derived components, usually in the form of polysaccharides [1]. Polymicrobial communities exist in the human oral cavity, nasal cavity, gastrointestinal (GI) tract, respiratory tract, and urogenital tract [2].

The GI tract and oral cavity are inhabited either permanently or temporarily by a large number of unique microbial species (approximately 600–1000) [1,3,4]. The great variety of microbes in the mucosa and the limited space available led to physical and chemical interactions over hundreds of years of evolution [5]. These interactions can take several forms. The first is synergism, where one microbe forms a niche for another microbe to inhabit or to infect. The second is predisposition, in which interaction between the host and a microbe predisposes another microbe to colonize. Third, antagonism or microbial interference describes host–microbe interactions that decrease or inhibit colonization by another microbe. Finally, addition is when two non-pathogenic organisms can cause infection only when combined [6].

Multispecies colonization of human tissue has been observed using microscopes. However, little is known about how multispecies interactions determine the extent, progress, and severity of human disease or about how the body reacts to polymicrobial versus monomicrobial infection [1,2]. Most infectious diseases were previously recognized to be monomicrobial, perhaps due to the use of culture-dependent techniques. However, using culture-independent community analysis methodologies, several diseases have been characterized as polymicrobial infections, such as oral cavity disease, otitis media, chronic infection in the cystic fibrosis lung, and diabetic foot wound infections. In those polymicrobial-related infections, severity and disease outcomes can be predicted from the microbial composition. Several measures may enhance surveillance of potential disease risk factors, such as epidemiologic identification and pyrosequencing of the microbial community when these diseases occur and study of the relationships between microbes. Preventive and treatment strategies must be continuously improved to fight polymicrobial diseases [1,7].

The availability of effective antimicrobial drugs is important for the successful treatment of infectious diseases [8]. However, increasing cases of multi-drug-resistant pathogens, the small selection of effective antimicrobials, and limitations to antimicrobial drug delivery hamper treatment of these diseases [8,9,10]. In this Review, we present the pharmacological approaches available and readily used to treat mono- and polymicrobial diseases and the challenges facing the field. We further discuss the potential use of novel natural product-derived active compounds with anti-polymicrobial properties that mainly play a role in the inhibition of biofilm formation. We also discuss several newly developed drug-delivery systems targeting intracellular and extracellular pathogenic microbes and their potential applications in the pharmacological treatment of polymicrobial diseases. These promising pharmaceutical technologies may provide important advances in the treatment of polymicrobial diseases.

## 2. Types of Polymicrobial Interactions 

Polymicrobial interactions can be broadly divided into two types: synergistic combinations of two or more microbes to initiate infection, termed polymicrobial diseases, or antagonistic interactions in which colonization by one microbe interferes with colonization by another [7]. Polymicrobial diseases are acute and chronic diseases caused by diverse combinations of bacteria, fungi, viruses, and/or parasites in a particular host (Table 1) [7]. In synergistic polymicrobial diseases, one microorganism creates a niche ideal for the infection and colonization by other, frequently pathogenic microorganisms. Human metapneumovirus and coronavirus, for instance, co-exist in patients with respiratory syncytial virus in bronchiolitis [11] and also with severe acute respiratory syndrome [12] and other respiratory infections [13]. 

Polymicrobial interactions may also take the form of interference, in which one microbe interferes with another to colonize or infect the host. Interference signature has been widely documented in invertebrates (e.g., antiviral protection of intracellular bacteria *Wolbachia* in *Drosophila melanogaster*) [14] and in vertebrates (e.g., co-infection of a human host with human immunodeficiency virus (HIV) and non-pathogenic human GB virus C) [15]. In the latter example, GB virus C can inhibit HIV replication, leading to the reduction in host mortality rate [15]. Furthermore, patients co-infected with HIV and GB virus C have higher baseline CD4+ T cell counts, a slower rate of decline of CD4+ T cells, and lower plasma levels of HIV RNA than HIV-positive patients without GB virus C co-infection [15]. 

This Review focuses on the occurrence and treatment of polymicrobial diseases, commonly triggered by a synergistic combination of two or more pathogens [7]. An example is the measles virus, a single-stranded, negative-sense, enveloped RNA virus that causes death mostly by suppressing the host immune response, thereby promoting the development of secondary bacterial infections [16,17]. It has been postulated that an immunosuppressive factor generated by measles virus-infected lymphoid cells might prevent the proliferation of antibody-producing B cells [16,18].

Some viruses of the family Retroviridae have been demonstrated to infect host immune cells, impairing antigen recognition and/or antibody production. With such immunosuppression signatures, HIV and closely related viruses are the best examples of “partner-in-crime” pathogens that lead to secondary bacterial infections [19,20]. Many patients with HIV-AIDS in Africa are also co-infected with *Mycobacterium tuberculosis* and malaria parasites [19,20,21,22] and with other bacteria, viruses, fungi, and protozoans [23,24]. Moreover, increasing cases of bladder and kidney infection have been reported in patients with human T-cell lymphotropic virus type I (HTLV-I), a retrovirus of the genus Deltaretrovirus [25]. Likewise, incidences of acute bronchitis, bladder and kidney infection, arthritis, and asthma have been shown to rise in patients infected with HTLV-II [25], another retrovirus.

In some cases of polymicrobial diseases, infection occurs in certain tissues or organs as a consequence of preliminary colonization by another microbe [1,7]. Initial colonization by one microbe might predispose the host to infection or colonization by a second microbe in the same sites [1,7]. For example, the destruction of respiratory epithelium by certain respiratory tract viruses, such as influenza virus and human respiratory syncytial virus, can promote secondary bacterial adhesion [26]. Respiratory tract viruses can also predispose a host to middle ear infections [27,28]. These viruses may cause bacterial superinfections, mainly by suppressing host immune response, or by increasing the expression of certain receptor-associated molecules that can facilitate bacterial attachment to the target tissues [29]. In the additive type of polymicrobial interactions, two or more nonpathogenic microorganisms synergistically colonize the host, resulting in bacteremia, which can manifest in various organs from soft tissue to lung, liver, and brain [7]. Detailed types of interaction and examples of combination of microorganisms involved in the polymicrobial diseases are presented in Table 1.

## 3. Targeting Polymicrobial Diseases: Current and Promising Approaches

Antimicrobial resistance is an ongoing and daunting issue nowadays, as this has put humans at greater risk of death due to untreatable infection [9]. Bacterial diseases, such as tuberculosis, salmonella, and pneumonia, have been shown to threaten global communities and this unfortunate situation have been reported to be rising as numerous bacteria are becoming tolerant to antibiotics [9,30]. The term “antimicrobial resistance” describes the resistance of viruses, bacteria, fungi, etc., to medicines, which were originally efficient in treating microbial infection [9,30]. To treat multiple infectious diseases and particularly to counter drug-resistant microbes, the utilization of newly discovered antimicrobial agents derived from natural products and microbial cultures present a potential approach in the treatment of infectious diseases. In this section, the use of traditional antibiotics and the potential utilization of natural products as promising antimicrobial candidates against polymicrobial infections are discussed.

### 3.1. Antibiotics: Pharmaceutical Arsenals in Polymicrobial Diseases

**Figure 1:**
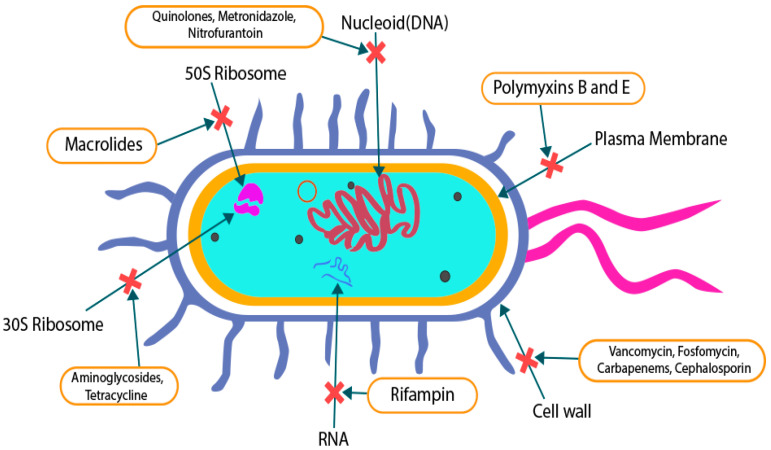
Sites of action for different types of antibiotics.

Most polymicrobial diseases are characterized by virus–bacterium or bacterium–bacterium interactions [1,7]. To treat these cases, we heavily rely on the availability of effective antimicrobial agents. Since most of the alarming polymicrobial diseases involve secondary bacterial infections, especially in the case of HIV-related immunodeficiency, we focus our Review on currently available antibacterial or antibiotic compounds. In general, antibiotics are defined as substances that inhibit the growth or selectively kill pathogenic bacteria [10]. Based on their clinical pharmacology properties, antibiotics can be divided into several categories [8]. In this Review, we present the most significant antibiotics based on their functions and sites, and mechanisms of action (Figure 1; Table 2).

#### 3.1.1. Cell-Wall Synthesis Inhibitors

The existence of polysaccharides in the cell wall of bacteria, commonly known as peptidoglycan, distinguishes it structurally from that of other species [31]. Vancomycin, beta-lactam drugs, and fosfomycin possess great therapeutic potency to interfere with bacterial cell-wall synthesis. Beta-lactam drugs include carbapenems, cephalosporins, penicillin derivatives, and monobactams. When beta-lactam drugs attach to penicillin-binding proteins (PBPs), which are essential for catalyzing the cross-linking of the peptidoglycan layer, cell-wall synthesis is disrupted [8,31]. Cell-wall hydrolysis then occurs, leading to the aggregation of peptidoglycan and impairment of cell-wall formation [8,31]. This further promotes peptidoglycan digestion and bacterial burst. Vancomycin and fosfomycin primarily act by targeting the d-Ala-d-Ala (D-alanyl-D-alanine) terminus and MurA (UDP-N-acetylglucosamine enolpyruvyl transferase), respectively, and disrupt the initial step of peptidoglycan synthesis [31,32,33].

#### 3.1.2. Membrane Function Inhibitors

The cytoplasmic membrane is vital to maintaining homeostatic regulation of physiological function in microbial cells [8,34]. It serves as a selective barrier for ions and selected macromolecules. Alteration of the membrane’s functions promotes the disproportion of essential ions and macromolecules, which contributes to the breakdown or destruction of the cell [34]. Polymyxins have long hydrophobic tails and are considered to have strong antibacterial properties. Polymyxins B and E are potent and widely used for therapeutic purposes. When polymyxins bind to the portion of lipopolysaccharide that exists on the outer surface of Gram-negative bacteria, the selective barrier function is impaired due to modification of the membrane structure [34]. This disrupts both the inner and outer membranes of the affected microbes [34,35]. 

#### 3.1.3. Protein Synthesis Inhibitors

Protein synthesis, both in humans and bacterial cells, is an essential task for cellular survival. The ribosome is the most important organelle in the translation of mRNA into protein [8,36]. Bacterial ribosomes are comprised of two subunits, the 30S and 50S [36], and differ structurally from human ribosomes, which have 40S and 60S subunits [37,38]. Bacterial ribosomes thus serve as a selective target to inhibit protein synthesis. Aminoglycosides and tetracyclines block the 30S subunit where macrolides target 50S to inhibit protein synthesis [39,40]. Aminoglycosides and tetracyclines function by preventing aminoacyl tRNAs from accessing the ribosome and binding with the 16S rRNA component of the 30S subunit, respectively, inhibiting protein production [39,40]. It has been suggested that aminoglycosides disturb all typical steps in protein biosynthesis, such as translation initiation, peptide bond elongation, etc. [39]. In contrast, macrolides operate effectively by either initiating or dislocating the peptidyl tRNAs, which prevents the peptidyl–transferase reaction from lengthening the local peptide chains [38]. 

#### 3.1.4. Nucleic Acid Synthesis Inhibitors

Nucleic acid synthesis is one of the most critical targets for antibiotics to treat communicable diseases [41]. To simplify classification, the antimicrobial agents in this class are subdivided into DNA and RNA inhibitors. RNA blockers interact with bacterial transcription by generating RNA transcripts of genetic material for further translation into proteins [41]. Rifampin, a popular RNA inhibitor, primarily targets the DNA-dependent RNA polymerase enzyme and impedes RNA elongation, which promotes bacterial cell death [42]. DNA synthesis is inhibited by quinolones, metronidazole, etc. By targeting the DNA gyrase, quinolones disrupt DNA replication, which leads to bacterial cell death [43]. In addition, metronidazole and nitrofurantoin are also associated with DNA biosynthesis blocking. These drugs mainly act on the anaerobic bacteria by producing metabolites that attach to the DNA strands and promoting bacterial cell rupture [34,36]. 

#### 3.1.5. Metabolic Pathways Inhibitors 

Sulfonamides and trimethoprim (TMP) are two metabolic pathway inhibitors used in combination (in the form of sulfamethoxazole (SMX)–TMP) to negatively impair bacterial folic acid metabolism synergistically [44]. TMP inhibits the action of the dihydrofolate reductase enzyme, and SMX inhibits the conversion of para-aminobenzoic acid into dihydropteroic acid by preventing the action of dihydropteroate synthetase. The action of both antibiotics prevents the synthesis of folic acid, producing their bacteriostatic effect [44].

### 3.2. Natural Products: Alternative Effort in Combating Polymicrobial Diseases

Antibiotics have been clinically shown to exert potent pharmacological activity against diverse bacterial pathogens. However, these drugs have been reported to cause multiple adverse effects on the human body [88]. Certain antibiotics have been associated with the increasing incidence of neurotoxicity in high-risk populations, such as the elderly and patients with renal insufficiency or prior central nervous system disease [89]. In recent years, natural products, including those derived from plants and microbes, have been seen as promising alternatives to antibiotics, mainly due to their potential novel mechanism(s) of action [90,91]. Our Review focuses on prospective natural products derived from plants (Table 3). 

#### 3.2.1. Essential Oils (EOs)

Essential oils (EOs) are some of the most effective natural products with broad antimicrobial activity, mainly via the suppression of growth and proliferation [92]. EOs have been demonstrated to treat bacterial infections [93]. Specific antimicrobial activities against *Pseudomonas*, *Salmonella*, *Staphylococcus*, and others have been reported for clove, cinnamon, and rosemary EOs [94]. Of these, the most potent is clove oil. The key components derived from *Melaleuca alternifolia*, such as *γ*-terpinene, terpinene-4-ol, and terpinene-4-ol, are used to inhibit the development of certain bacteria in the human body, like *Escherichia coli*, *Staphylococcus epidermidis*, *Staphylococcus aureus, Streptococcus pneumonia,* and *Pseudomonas aeruginosa* [95,96]. These bioactive compounds provide antibacterial action via the inhibition of bacterial DNA and protein synthesis [97].

EOs and their derivatives possess a high degree of efficacy against fungal diseases, with the most impactful ones obtained from spices [98]. Experimental testing of crude extract of garlic and clove demonstrated the inhibition of fungal growth of some *Candida* species [98]. Oregano and thyme EOs were the strongest inhibitors of fungi; they contain phenolic compounds, such as thymol and carvacrol, which can impair the function of fungal cell membranes [99]. In addition to their antibacterial and antifungal activities, EOs have activity against certain viral pathogens. Crude extracts from *Origanum acutidens* and callus cultures have antiviral properties. EO from *Salvia fruticosa* has a cytotoxic effect against African green monkey kidney cells and enhanced virucide activity against human herpes simplex virus (HSV)-1 [100,101,102,103].

#### 3.2.2. Alkaloids

Alkaloids from natural sources are secondary metabolites that possess strong therapeutic activities. One study investigated the potential effect of alkaloids from the leaves of *Vernonia adoensis* and *Callistemon citrinus* on the growth, proliferation, and efflux pump function of *P. aeruginosa* and *S. aureus* [104]. *Callistemon citrinus*-derived alkaloids have high therapeutic potency against *S. aureus* (minimal inhibitory concentration (MIC) of 0.0025 mg/mL) [104]. In addition, the alkaloid extract possesses bacteriostatic effects on *P. aeruginosa.* The strong bacteriostatic effects of alkaloid extract differ in contrast to *P. aeruginosa* because of the pathogen’s thick outer membrane that is very hydrophobic, which may provide a permeability barrier to the extract [104]. Moreover, alkaloids obtained from the fruits of *Cucumis metuliferus* are highly efficacious against infectious bursal disease virus [105], and natural alkaloids are also effective against influenza virus. Naturally occurring alkaloids impair the synthesis of viral proteins on the different stages of viral replication and influence modifications in the other groups of alkaloids. The chemical modifications in the nature of the various alkaloid derivatives have also shown their enhanced effects on influenza viruses [106].

#### 3.2.3. Terpenes

The usage of terpenes and their derivative products against microbial disorders is popular due to their large potential effect. Terpenes have been demonstrated to work well against multi-drug-resistant bacteria [107]. For example, the increasing prevalence and growth of *S. aureus* biofilm can be disrupted by terpenes [108]. Furthermore, terpenes possess a potent synergistic effect with certain antibiotics, decreasing the development of antibiotic-resistant bacteria. For example, synergistic effects have been established between terpene and gentamicin (antibiotic) when both are applied together, and the consequences have been noted in the significant decrease in microbial growth [109]. Terpene and its byproducts have also been successfully tested against the herpes simplex virus. These compounds significantly inhibit the action of viral DNA polymerase during the production of new viral progenies [110].

#### 3.2.4. Phenolic Compounds

Phenolic compounds are important secondary metabolites that can be collected from various natural sources, mainly plants, and possess a large number of bioactive properties. These compounds have been shown to constrain the development of foodborne contagious agents, promoting their broad application as a research tool in the field of food science [111,112,113]. Numerous types of bacteria can induce severe food poisoning [114]. Phenolic compounds have been reported to prevent nutrition decay due to pathogens and/or toxins [112,115]. Additionally, phenolic byproducts are also potent against the *Xylella fastidiosa* bacterium, which may affect commercially valuable crops and plants. Crops with enhanced tolerance to the *X. fastidiosa*-induced disease will effectively be developed by growing the endogenous anti-xylella phenolic concentration rather than adding novel antibacterial agents [116].

Phenolic acids, tannins, coumarins, and quinines are among the phenolic compounds commonly extracted from medicinal plants and herbs [117,118,119]. Phenolic compounds such as curcumin, carvacrol, and thymol have been shown to inhibit the growth of *Candida albicans*, the causative agent of candidiasis. The suppressive effects of phenolic compounds are attributed to their molecular structure: a non-polar portion that enables them to move through the membranes and a hydroxyl group coupled with a delocalized electron system that offers the molecules an acidic characteristic, leading to the instability of the fungal cell membrane [120]. Furthermore, thymol, carvacrol, and eugenol have also been demonstrated to yield antifungal activity against *Aspergillus* sp. [121]. Phenolic compounds are also well known for their antiparasitic activity. For instance, *Guazuma ulmifolia* Lam., a plant that belongs to the *Malvaceae* family, is a valuable source of quercetin that yields pharmacological activity against some parasites, such as *Leishmania braziliensis, L. infantum*, *Trypanosoma cruzi* and others via its cytotoxic action. The cytotoxicity that is determined in ethanol extract makes it is a potential candidate to have antineoplastic activity in tumor cells [122].

#### 3.2.5. Quinones

Owing to their potent antimicrobial properties, quinones are considered one of the most important natural products in the fight against bacteria and parasites [123,124]. Several quinones have been shown to yield antimicrobial activities: thymoquinone, plumbagin, and embelin. *Nigella sativa* plant seeds, a key source of thymoquinone, have the potential to inhibit the growth of microbial pathogens [125]. The utilization of thymoquinone with MIC of 8–64 μg/mL suppresses the growth of Gram-positive bacteria [126]. In addition, thymoquinone has been reported to be effective for certain bacterial pathogens with biofilm formation ability, such as *P. aeruginosa* and *S. aureus* [127]. Thymoquinone was successfully shown to reduce the virulence of *Fasciola gigantica*, the causative agent of fasciolosis in the tropical regions [128]. An in vitro study concluded that thymoquinone was able to reduce the expression of glutathione-S-transferase (GST) and superoxide dismutase (SOD) thus inhibited the detoxication ability of *F. gigantica* [128]. Two other studies demonstrated that the antiparasitic activity of thymoquinone against *Encephalitozoon intestinalis* [129], *Entamoeba histolica* [130], *L. tropica* and *L. infantum* [131], all of which are clinically-important to humans. Thymoquinone continues to undergo either nonenzymatic or enzymatic semiquinone radical redox cycling to cause superoxide anion radicals. Reactive oxygen species (ROS)-triggered oxidative stress irreversibly destroys the bacterial proteins and DNA [132].

*Plumbago* spp., naturally occurring plants, are the source of the phenolic compound plumbagin. Plumbagin possesses potential therapeutic properties against diverse microbes, including mycobacteria [133]. Plumbagin isolated from *P. scandens* yielded antibacterial effect against both *S. aureus* and *C. albicans* with MICs of 1.56 and 0.78, respectively [134]. Another source of plumbagin is *P. zeylanica*, which is highly efficacious against *Mycobacterium xenopi*, *M. smegmatis*, and *M. chelonae* [123,133]. Plumbagin demonstrated fasciolicidal effect against immature stages of *F. gigantica* parasites [135] and using both in vitro and in vivo model systems, a study demonstrated the antimalarial activity of plumbagin at a dose as low as 25 mg/kg body weight [136]. Another quinone is embelin, a compound present in *Embelia ribes* that is effective against pathogenic bacteria and fungi [137,138]. *Oxalis erythrorhiza*, a source of embelin, is particularly effective against *Trichophyton mentagrophytes*, *T. rubrum*, and *Microsporum canis* with MICs of 50–100 μg/mL [123,139]. Studies reported that embelin displayed bacteriostatic and bactericidal activities against Gram-negative and Gram-positive bacteria, respectively [123,140]. Embelin has also been shown to yield a dose-dependent in vivo antimalarial activity at doses from 100 to 400 mg/kg body weight (BW) [141]. In addition to that, embelin also demonstrated a potent anthelmintic activity against hookworm *Necator americanus* larva (in vitro) and *Hymenolepis nana* (in vivo) [142].

#### 3.2.6. Flavonoids and Its Derivatives

Flavonoids are available in the entire world and are collected from a variety of plant parts, such as seeds, stems, flowers, and fruit [143,144]. These natural products have a number of therapeutic features, including anti-inflammatory, antioxidant, and antimicrobial activities [143,145]. As a complement to antibiotics, flavonoids have come to researchers’ attention due to their prospective use against antibiotic-resistant bacteria [146]. Flavonoids have been reported to have various modes of action to yield their antimicrobial activities. Inhibition of DNA synthesis in *Proteus vulgaris* and RNA synthesis in *S. aureus* is induced by flavonoids [144,147]. Several research groups have conducted laboratory trials to investigate whether flavonoids can act as bactericidal or bacteriostatic agents. Using epigallocatechin and 3-O-octanoyl-(+) catechin as examples of flavonoids, two independent research groups demonstrated the bactericidal activities of flavonoids against antibiotic-resistant *S. aureus* [148,149].

In addition to their antibacterial activity, flavonoids also demonstrate potent antifungal actions against *Aspergillus*, *Candida*, *Pneumocystis*, and *Cryptococcus* [150]. In general, flavonoids exert their antifungal actions by several mechanisms, such as inhibition of cell wall formation, plasma membrane destruction, mitochondrial dysfunction, interruption of cell proliferation, and inhibition of DNA replication [150]. While flavonoids have shown great antibacterial and antifungal potential, research on the antiviral activities of flavonoids is still in its infancy. Preliminary in vitro screening experiments have shown promising antiviral activities of flavonoids against several viruses, such as murine norovirus and feline calicivirus [151] as well as HIV-1 [152].

## 4. Potential Role of Drug-Delivery Approaches in the Treatment of Polymicrobial Diseases

Despite the significant achievement accomplished by the application of antibiotics against polymicrobial infectious diseases, bacterial infections remain a significant problem worldwide [163,164,165]. Elimination of numerous bacterial infectious diseases has been hampered by the complicated mechanisms of the bacterial pathogen in disrupting the immune system of its host. Furthermore, barriers to delivery prevent antibiotics from reaching the sites of infection [165,166,167]. On the other hand, several potent antimicrobial agents, such as fluoroquinolones, aminoglycosides and other antibiotics, produce severe side effects and are reserved only for serious infections [165,168,169]. Essentially, the presence of biofilms in the area of infections has led to an increase in resistance of numerous bacterial to antimicrobial agents [9,170]. This emphasizes the requirement for alternative and efficient antimicrobial approaches to overcome challenges in the delivery systems.

Recently, nanotechnology, especially nanoparticles (NPs), has been applied to drug delivery [165,171,172,173,174,175]. Several NP approaches, including polymeric NPs, liposomes, metal NPs and lipid NPs, have been widely investigated as delivery platforms for antimicrobial agents. Drug molecules incorporated into NPs via several encapsulation methods, including physical encapsulation, chemical conjugation or adsorption, exhibit an enhanced pharmacokinetic profile and therapeutic index in comparison with their free drug equivalents [176]. Other benefits of nanocarrier delivery methods, such as enhancement in the solubility of drugs, extended systemic circulation, specific drug targeting, controlled and sustained release manners, and simultaneous delivery of numerous drugs, have also been confirmed in many studies [165,173,177,178,179,180,181,182,183,184,185,186,187,188]. Consequently, the application of several antibiotic-loaded NP delivery approaches has been accepted for clinical studies in the management of numerous bacterial infections. Additionally, numerous NP formulations containing antimicrobial agents are presently under investigation in several stages of pre-clinical and clinical studies [175].

### 4.1. Polymeric NPs

Polymeric NPs have been used in the area of controlled release for over 25 years for both systemic and local administration of drugs, and, importantly, have been used in clinical studies. This type of NP is categorized into nanometric assemblies in the range of 10–1000 nm, offering physicochemical characteristics, including small size and large surface area, as well as different surface charge properties, which make them significant release systems. Essentially, the properties of polymeric NPs can be easily adjusted by varying their composition and functionalizing their surface [185,186,188,189,190,191]. 

**Figure 2:**
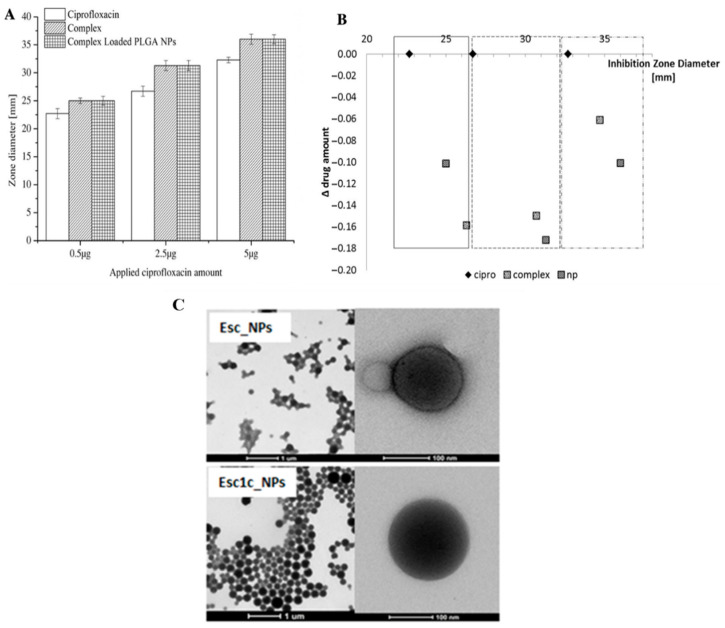
The zone of inhibition of ciprofloxacin and its nanoparticle (NP) formulation against *P. aeruginosa* (**A**) and illustrative results after normalization to released amount of ciprofloxacin NPs compared to free ciprofloxacin (**B**) [192]. TEM images of the Esc NPs (**C**) [184]. All figures are reprinted with permission of the publishers.

Poly (lactic-co-glycolic acid) (PLGA) NPs are one of the most extensively investigated types of NP for the delivery and the application of antimicrobial agents. PLGA is a biodegradable and biocompatible polymer. An in vitro study evaluated the antibacterial activity of rifampin loaded into PLGA NPs for 24 h using zone-of-inhibition study performed in agar plates. The results showed that rifampin-loaded PLGA NPs exhibited higher bactericidal activity against *S. aureus* and methicillin-resistant *S. aureus* (MRSA), and *Bacillus subtilis* in comparison with the free drug (Figure 2A,B). This might be because the encapsulation of rifampin into PLGA NPs could potentially improve the penetration of rifampin into bacterial cells and targeted delivery of rifampin to the area of action [192]. 

Ciprofloxacin was formulated into PLGA NPs for pulmonary delivery as a treatment option for cystic fibrosis. In this study, Türeli et al. (2017), by utilizing the MicroJet Reactor (MJR), were able to obtain NPs with a particle size of 190.4 ± 28.6 nm with 0.089 polydispersity index (PDI). Furthermore, the encapsulation efficiency of ciprofloxacin was 79%. The formulation of ciprofloxacin into NPs was able to control the release in medium independent including phosphate-buffered saline (PBS), PBS + 0.2% Tween 80, and simulated lung fluid. Importantly, with regard to MIC, the NPs were found to be non-toxic, evaluated by cytotoxicity assays with Calu-3 cells and CF bronchial epithelial cells (CFBE41o^−^). This study showed that the antibacterial activity of ciprofloxacin increased significantly when formulated in PLGA NPs. Moreover, the NPs were stable in the mucus environment, and the turbidity of mucus decreased when incubated with NPs. Therefore, this approach could be a promising delivery system of ciprofloxacin against *P. aeruginosa* infections of the lung in cystic fibrosis [183].

With the same purpose, Casciaro et al. (2019) developed PLGA NPs to deliver antimicrobial peptides (AMPs) (Esc peptide) to the lung (Figure 2C). The NPs were prepared using the solvent diffusion–emulsion method. The NP formulation was also able to enhance the antimicrobial activity of AMPs. Interestingly, in the in vivo study, Esc-peptide-loaded NPs were administered intratracheally in a mouse model of *P. aeruginosa* lung infection, and the results showed that after 6 h of administration, there was a reduction of three logarithms in *P. aeruginosa* bioburden [184].

In addition to PLGA, poly(ε-caprolactone) (PCL) has also been explored as a suitable polymer to deliver antimicrobial agents to infection sites. A study described the synthesis of bacterial lipase-sensitive PCL nanogel for selective delivery of antibiotics to an infection area. Significantly, research findings exhibited that the release of drugs from this delivery system only occurred in the presence of a lipase enzyme produced by *S. aureus* strains [184]. Using this reason, a study developed carvacrol loaded into PCL NPs for improved antimicrobial activity against polymicrobial biofilm-infected wounds [193,194,195]. The encapsulation of carvacrol in PCL NPs could potentially increase antimicrobial activity 2–4-fold against several strains of *S. aureus* and *P. aeruginosa*. To prove the selectivity of this approach, the release study was performed in the presence and the absence of various bacterial cultures. The results showed that the release of carvacrol was significantly enhanced in the presence of *S. aureus* and *P. aeruginosa*, and the enhancement of antibiofilm activity was also confirmed in an ex vivo biofilm model when delivered intradermally using a microneedle delivery systems. 

Similarly, a study developed different types of polymeric NPs, namely PLGA NPs, PCL NPs, chitosan NPs, PLGA-decorated chitosan NPs, and PCL-decorated chitosan NPs, to encapsulate doxycycline for enhanced antibiofilm activity in polymicrobial-infected wound models [196]. The results showed that the release of doxycycline was affected by the selection of the polymers. In addition to PCL, PLGA [197] and chitosan [198] have also been reported to be hydrolyzed by lipase enzyme produced by *S. aureus* and *P. aeruginosa*. It was found that the release of doxycycline was higher from PCL NPs than from PLGA NPs. This might be because the PCL chains are more flexible than PLGA chains [199], resulting in rapid hydrolysis by lipase esterase produced by *S. aureus* and *P. aeruginosa* in the release media. Importantly, when delivered by dissolving microneedles intradermally, the antimicrobial activity of doxycycline increased significantly in an ex vivo biofilm model in porcine skin. Several studies have shown the effectiveness of microneedles to deliver antimicrobial agents to the skin [200,201].

A recent study formulated clarithromycin into PLGA NPs to target intracellular *S. aureus* and *M. abscessus* [181]. After encapsulation into PLGA, clarithromycin could reduce 1000 times the viability of intracellular *S. aureus* and *M. abscessus* with 70–80% killing percentage, when compared to free clarithromycin. In their study, the antimicrobial activity of clarithromycin-loaded NPs was also evaluated in vivo, using murine and zebrafish models. The in vivo studies showed that the permeability of clarithromycin across Calu-3 monolayers was improved in NP formulations compared with free clarithromycin, suggesting enhanced delivery to sub-epithelial tissues.

### 4.2. Metal NPs

Metal NPs have been reported to have antimicrobial activity for decades. However, the toxicity issue of metallic NPs has not been fully understood. Several studies have shown that these NPs could be accumulated in tissues and organs [178]. In terms of immunogenicity, research related to the physical–chemical characteristics of metallic NPs and their possible impacts on the immune system is limited. More investigations are required to obtain a more detailed understanding of these potential effects [202]. 

Metallic NPs from cerium oxide (CeO_2_) have been investigated by Selvaraj et al. (2015) for alternative therapy of severe sepsis. The size of NPs was found to be 140 ± 53 nm, and the antimicrobial activity study showed that these NPs were able to inhibit the growth of *E. coli* (ATCC 35150) and *S*. *aureus* (ATCC 29213) in concentrations of 50 and 100 mg/mL, respectively. An in vivo study in a polymicrobial sepsis model in rats showed that the administration of CeO_2_ NPs intravenously increased the survival of infected rats from 20% to 90% after 48 h. Administration of these NPs also decreased organ damage in infected rats [203]. 

A recent study prepared gold NPs synthesized using phytoconstituent from the extract of *Acalypha indica*. The NPs were approximately 20 nm in size. The gold NPs possessed strong antibacterial activity against *S. epidermidis* and *E. coli* bacterial strains [182]. 

A study developed gold NPs prepared using the hydrogen-producing hyperthermophilic bacterial strain *Caldicellulosiruptor changbaiensis*. The NPs obtained had remarkable antibacterial activity against both Gram-positive and Gram-negative bacteria. In this study, the NPs also possessed antibiofilm activity in vitro. Furthermore, the authors developed biofilm models in BALB/c mice, and the administration of gold NPs intravenously was able to eradicate bacterial biofilms [204].

Another type of metallic NP, silver NPs, has shown a wide range of antimicrobial activity compared to other metallic NPs. Using glycolic acid as a reducing agent, Kumar et al. (2020) synthesized silver NPs as antibacterial compounds for the potential treatment of skin infections. The NPs were found to be 18 ± 2 nm in size. Importantly, NPs obtained showed excellent antimicrobial activities against *Klebsiella pneumoniae*, *E. coli*, *S. marcescens*, *P. aeruginosa*, *Serratia marcescens*, *Salmonella typhimurium*, *S. aureus* and *Staphylococcus epidermidis*. Moreover, biocompatibility and cytotoxicity studies performed in skin cell lines (HaCaT) showed that the silver NPs were non-toxic [205]. 

**Figure 3:**
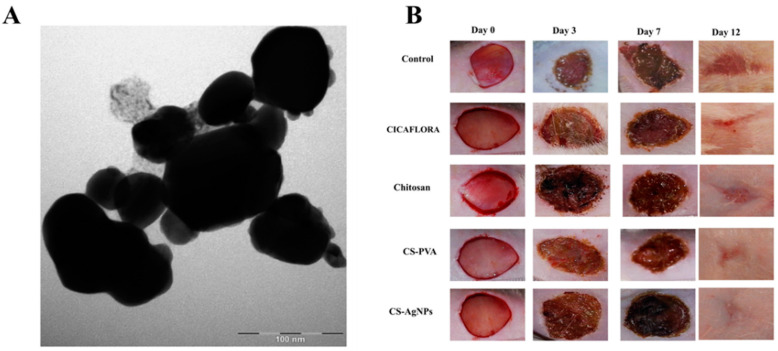
TEM images of chitosan (CS)–silver NPs (**A**) and illustrative images of macroscopic appearance of the in vivo wounds healing studies after the administration of CS–Ag NPs gels compared to different type of treatments (**B**) [177]. All figures are reprinted with permission of the publishers.

Hajji et al. (2019) prepared chitosan–PVA–silver NPs using the green technique. In their study, chitosan and PVA were used as stabilizers (Figure 3A). Particle size analysis showed that NPs were consistently distributed in the matrix with diameters of 190–200 nm. The in vitro antibacterial activities revealed that these NPs were active against four Gram-negative bacteria, *E. coli*, *P. aeruginosa*, *Salmonella enterica* serovar Typhi, and four Gram-positive bacteria, *S. aureus*, *Micrococcus luteus*, *Bacillus cereus*, and *Enterococcus faecalis*. Furthermore, the silver NPs showed low cytotoxicity at concentrations of 5–200 μg/mL in Chinese Hamster Ovary (CHO-K1) cells. Interestingly, the authors also evaluated in vivo wound healing activity in Wistar rats (Figure 3B). The results showed that the NPs could stimulate wound healing, due to their antibacterial and antioxidant synergistic activities, making them suitable for wound care treatment [177].

**Figure 4:**
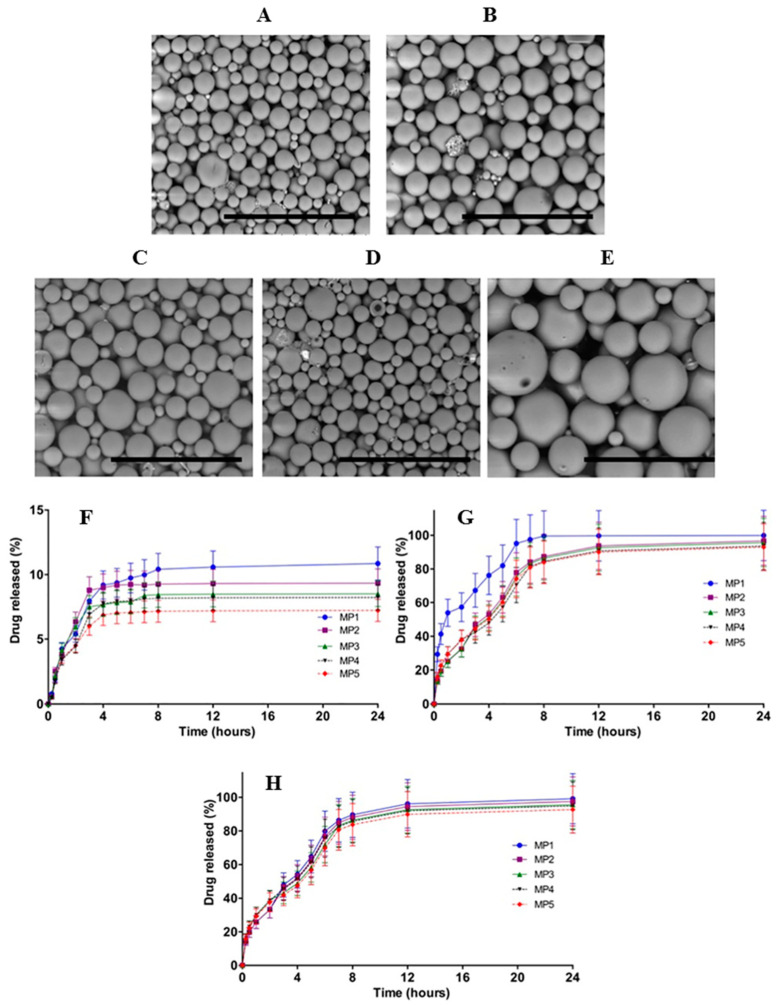
SEM images of different type of microparticles (MPs) laden with silver NPs (**A**–**E**). In vitro release of silver NPs from MPs without (**F**) and with the presence of *Staphylococcus aureus* (**G**) and *Pseudomonas aeruginosa* (**H**) [206]. All figures are reprinted with permission of the publishers.

Recently, Permana et al. (2020) developed bacterial responsive microparticles containing silver NPs synthesized from green tea extract (Figure 4). The selective microparticles were developed to avoid the toxicity issue of silver NPs in the non-infected area. The microparticles were prepared from PCL and chitosan. The results showed that incorporation of silver NPs into this approach was able to avoid unnecessary release of silver NPs without the presence of bacteria. Furthermore, using dissolving microneedles (MNs), this combination approach was found to be effective in the treatment of bacterial biofilm infection (*S. aureus* and *P. aeruginosa*) in ex vivo infection models in rat skin [206]. 

### 4.3. Liposomes

Liposomes were developed in the early 1960s and, due to their lipid bilayer, were initially applied in research to model the plasma membrane [207]. Thanks to their unique characteristics, including low toxicity, nanometric size, high biocompatibility and biodegradability, capability to traverse membranes, and ability to load hydrophobic and hydrophilic compounds, liposomes have been extensively applied in the delivery of various drugs, including antimicrobial compounds [208]. 

Meers et al. (2008) formulated amikacin into a liposomal delivery system for improved treatment of chronic pulmonary infection of *P. aeruginosa*. The liposomes were prepared using 1,2-dipalmitoyl-*sn*-glycerol-3-phosphocholine (DPPC) and cholesterol. The chronic pulmonary infection model was developed in female rats after intratracheal administration of *P. aeruginosa*. The results showed that amikacin liposomal exhibited a better in vivo antimicrobial activity against *P. aeruginosa* in comparison with the free amikacin. Accordingly, the formulation of liposomal of amikacin could potentially be used as an alternative therapy for chronic pulmonary infections [209].

Polymyxin B-related colistin was reported to have antimicrobial activity against several Gram-negative bacilli. However, the cytotoxicity and low permeability of the drug limited its utilization [210]. In order to increase the effectiveness of this drug, while avoiding these drawbacks, Li et al. (2016) formulated colistin in the liposome delivery system. They investigated its bioavailability and antimicrobial activity against *E. coli*. In their formulation, to enhance the permeability and the electrostatic interaction of the phospholipid bilayer of bacteria with colistin, the liposomes were functionalized with sodium cholesteryl sulfate. The findings revealed that the incorporation of colistin into liposomes could potentially decrease colistin toxicity, increase the concentration and circulation time of colistin in the bloodstream, improve the efficiency of colistin to localize to the infectious targets, and improve its antimicrobial activity [211].

Most recently, Cai et al. (2020) developed a liposome formulation containing vancomycin and investigated its use in combination with a polylactide (PLA) composite fracture fixator. In this study, the cationic liposome was prepared, and the incorporation of vancomycin could potentially increase antibacterial activity against *S. aureus* and *E. coli* and sustain the release of vancomycin from the lipid matrix, as well as decrease in vivo toxicity of vancomycin. More importantly, the combination of vancomycin liposome with PLA composite internal fixator was found to possess outstanding osteogenic activity and antimicrobial capabilities in both in vitro and in vivo studies in a fracture model in adult male C57BL/6 mice. These results offer an alternative antimicrobial material for application in the clinical fracture field [212].

### 4.4. Solid Lipid NPs

Solid lipid NPs (SLNs) were initially investigated in the 1990s, and their use has been expanded to deliver numerous types of drugs, including antimicrobial agents. SLNs contain a core of solid lipid enclosed by a single lipid layer creating an outer covering. In the formulation, different types of solid lipids can be used, such as triglycerides, fatty acids, steroids, and waxes [213,214,215,216]. The use of this system by the pharmaceutical industry has grown, as it provides greater drug solubility, prolonged release, low toxicity and greater protection from drug degradation [179,187].

A study incorporated ofloxacin into hybrid SLNs by combining chitosan as a cationic biopolymer having antimicrobial activity and eugenol as a phenolic agent interfering with bacterial quorum sensing into a lipid myristic myristate matrix using a hot homogenization/ultrasonication technique [217]. The SLNs developed were found to be approximately 300 nm in diameter, and the release of ofloxacin was sustained for 24 h. Importantly, the ofloxacin-loaded SLNs showed improved antimicrobial activity against *P. aeruginosa* and *S. aureus*. The MIC of ofloxacin-loaded SLNs was 6.1–16.1 times lower than that of free ofloxacin, indicating improved antimicrobial activity. Furthermore, it was found that the SLNs could interrelate with the cell membrane of bacteria tested. Importantly, the toxicity study revealed no toxicity of the formulation in human cell models (A549 and Wi-38) following exposure for 24 h and 48 h. Finally, the in vivo study showed that the inhalation administration of ofloxacin-loaded SLNs was able to deliver ofloxacin to the lungs at a therapeutic concentration [217]. 

Kalhapure et al. (2017) synthesized a cleavable acid lipid to formulate pH-responsive SLNs to deliver vancomycin specifically to infection sites with acidic environments. The SLNs were 132.9 ± 9.1 nm in size with an encapsulation efficiency of 57.80 ± 1.1%. To evaluate the pH-sensitive release of vancomycin from the lipid matrix, a release study at different pHs was carried out, showing that the release of vancomycin at pH 6.5 (pH at infection site) was significantly higher compared to the release at pH 7.4 (normal physiology pH). Importantly, the antimicrobial activities of vancomycin in SLN formulations against methicillin-susceptible *S. aureus* (MSSA) and methicillin-resistant *S. aureus* (MRSA) were also significantly higher at pH 6.5 than pH 7.4. Finally, an in vivo study in an infection model in mice revealed that the viability of MRSA in the skin was significantly reduced following the administration of SLNs laden with vancomycin. Moreover, no inflammation was found in the skin of mice after treatment with SLNs, showing the safety of this approach [180].

Permana et al. (2019) developed SLNs to deliver antifilariasis drugs, doxycycline, diethylcarbamazine, and albendazole, separately to infection sites in the lymphatic system. The SLNs were prepared using glyceryl monostearate and stabilized with Tween 80. The SLNs were less than 100 nm, acceptable for lymphatic targeting. In an in vivo study, the drugs could be delivered using dissolving MNs to the lymph nodes of rats effectively, when compared to oral administration of free drugs as standard therapy for antifilariasis drugs, offering an efficient treatment for lymphatic filariasis [218]. 

## 5. Other Potential Approaches in the Treatment and/or Prevention of Polymicrobial Diseases

The unsuccessful treatment of polymicrobial infections sometimes can be attributed to the reduction in antimicrobials’ efficacy [219,220,221]. For example, *P. aeruginosa* exoproduct protect *S. aureus* against vancomycin, a drug used for treatment of methicillin-resistant *S. aureus* in cystic fibrosis patients [221]. *P. aeruginosa* exoproduct 4-hydroxy-2-heptylquinoline-N-oxide (HQNO) was also reported to protect *S. aureus* from tobramycin [222]. In addition, the failure of polymicrobial infections therapy is also caused by the formation of biofilms that protect microbes from antimicrobial drugs and host defense factors. For instance, fluconazole is not effective against *C. albicans*, due to the protection of *S. epidermidis* by releasing extracellular polymeric substance (EPS) that forms a biofilm [223]. In the biofilm, plasmid-mediated transfer of antibiotic-resistant gene(s) from one to other bacteria may also occur, leading to the escalation of antibiotic-resistant strains [224]. To prevent catastrophic consequences of polymicrobial diseases, novel approaches targeting biofilm and multiple pathogenic microbes are urgently needed.

Biofilm formation is critical for in the emergence of polymicrobial diseases. Examples of biofilm-associated polymicrobial diseases are infections in the oral cavity, inner ear, lung, urinary tract, and in wounds. Of these, most are due to the introduction of medical device to the hospitalized patients [225]. Biofilm has been defined as a complex three-dimensional structure comprising microbial communities that are surface-adherent and enclosed in a protective layer of exopolymeric substance [226]. Although myriad combinations of microorganisms can be hypothetically grown using the available in vitro and in vivo models, the most frequently documented interactions that lead to the formation of biofilm in the clinical settings are either bacteria–bacteria or fungi–bacteria [225,226]. For example, the concurrent presence of *C. albicans* and *E. coli* or *S. aureus* on various medical devices, such as endotracheal tubes and urinary catheters, are commonly reported [227,228]. In addition, another fungi–bacteria combination of *A. fumigatus* and *P. aeruginosa* has been reported to harbor the lungs of cystic fibrosis patients [229].

Biofilm formation is one of important virulence mechanisms used by numerous medically important bacterial and/or fungal pathogens. Therefore, biofilm remediation either by inhibition and/or dispersal of the targeted biofilm have been considered as potential strategies to treat polymicrobial diseases [226]. This can be achieved by inhibition of quorum sensing to prevent microbial cell-to-cell communication, which leads to the inhibition of biofilm formation. One of methods to do so is by the use of small molecule biofilm inhibitors, such as phenols, imidazole, furanone, indole, bromopyrrole, and other or biofilm dispersal agents. However, the use of biofilm dispersal agents remains challenging since the disperser cells have to be targeted immediately to prevent infection on another sites. The combination of biofilm dispersal agents shall be combined with the use of appropriate antibiotics [226]. Alternatively, the utilization of antimicrobials such as antimicrobial peptide LL-37 [230], and oritavancin [231,232] to target and eradicate biofilm-residing cells, defined as biofilm eradication agents (BEAs), is a possible standalone treatment that can be used. While this approach seems promising, it remains to be seen whether the use of BEA will work well in clinical settings. In the end, prevention of bacterial adherence and/or biofilm formation on the implanted medical devices and biomaterials by the coating of these materials with potent antimicrobial agents, for example silver nanoparticles, is one of methods that can be taken.

In addition to the drug delivery system, there are new pharmaceutical approaches to coat medical materials with antibiotics to avoid hospital-acquired infections, which also use polymers to prolong the drug release. Wang et al. (2017) developed a nanostructured surface containing nanopillars with various interpillar spacing on a new orthopedic implant chemistry, poly-ether-ketone-ketone (PEKK) [233]. In their study, following a 5-day evaluation, more than 37% of *S. epidermidis* viability on the PEKK surface was decreased in comparison with the orthopedic industry standard PEKK. Importantly, after one day of treatment, 28% of *P. aeruginosa* attachment was removed from PEKK surface and more than 50% of bacterial population was eradicated compared to the standard PEKK [233]. Furthermore, García-Arnáez et al. (2019) fabricated metallic bone implants coated with organic–inorganic hybrid coating materials. In this study, two bactericide agents widely applied to prevent bacterial infections, namely octenidine dihydrochloride and chlorhexidine diacetate were used to dope the coating materials. The results showed that the coating possessed sufficient mechanical and chemical properties and, therefore, this coating was suitable to be applied in the implant materials. Importantly, they showed antibacterial activity and did not show any toxicity in the human osteoblasts [234].

Kim et al. (2017) developed an implantable medical device based a polydimethylsiloxane (PDMS) film coated with AgNPs. The AgNPs attached in the films were evaluated for UV–vis spectroscopy, scattering electron microscope and induced coupled mass spectroscopy. The concentration of AgNPs was found to be about less than 0.05% of the weight of PDMS. Although the concentration was low, it was found that the attachment of AgNPs on the surface of the film was able to decrease the burden of *E. coli* with values of log_10_ 4.8 and *S. aureus* with values of log_10_ 5.7. Importantly, the coating process did not result in significant cytotoxicity [235]. With respect to the development of medical devices coated with sustained release material, Sikder et al. (2018) coated titanium alloy (Ti6Al4V) substrate with single-phase silver-doped antibacterial calcium deficient hydroxyapatite (CDHA) with a sustained release profile. These medical devices showed antibacterial activity against *E. coli* and *S. aureus*. Furthermore, they were found to be cyto-compatible with MC3T3 pre-osteoblasts cells. Importantly, the release of Ag+ was controlled and sustained for 14 days [236]. These methods have been shown to have potential in the mitigation of growth of diverse pathogens but mostly at the monomicrobial state. It would be interesting to see whether these approaches can prevent the rise of medical equipment-related polymicrobial infections in the future.

In addition to the use of potent antimicrobial and antibiofilm substances, there are several approaches such as the application of bacteriophage to combat bacterial pathogens or the clustered, regularly interspaced short palindromic repeats (CRISPR)-mediated gene silencing that has been considered promising to inhibit the formation of biofilm. Bacteriophages are ubiquitous in the biosphere, where bacteria survive [237]. A bacteriophage, or simply a phage, is a virus that targets certain bacteria as host for its propagation [238]. It can be categorized on the basis of its mode of action, i.e., lytic and lysogenic bacteriophages. Lytic bacteriophages, for example the T4 phage, follow the lytic cycle pathway which culminates in the immediate destruction of the host cell upon phage replication [238]. In contrast, temperate bacteriophages such as phage lambda of *E. coli* do not lyse its host immediately but integrate its genome to the bacterial host DNA and remain dormant until a certain condition is achieved, for example nutrient deprivation, to trigger their propagation [238]. Both types can be used in bacteriophage therapy as a means to combat bacterial pathogens.

Bacteriophage therapy has been suggested as an alternative to antibiotics in the management of infectious diseases with polymicrobial etiologies [1]. With their high specificity, bacteriophages may target specific bacterial pathogens, leaving mammalian cells and/or normal flora remain unharmed [239]. In addition, bacteriophages can exert potent enzymatic activity against certain bacterial exopolymeric compounds via the expression of depolymerases [1], thus they might be effective in targeting biofilm-associated pathogens. The biofilm clearance properties of bacteriophage were demonstrated by several studies including the ones carried out by Cerca et al. (2007) for the significant reduction in planktonic and biofilm cultures of *S. epidermidis* by bacteriophage K [240] and Glonti et al. (2010) for the breakage of the *P. aeruginosa* polysaccharide alginate matrix by alginase of bacteriophage PT-6 [241]. While this approach is promising, the clinical efficacy of bacteriophage therapy remains limited in humans. Further research and clinical trials are warranted to advance the bacteriophage therapy into mainstream therapeutical approaches in combating biofilm-associated polymicrobial pathogens.

Another novel approach that can be harnessed to tackle the biofilm-related issues is the use of a clustered, regularly interspaced short palindromic repeats (CRISPR)-mediated method. Studies have revealed the potential use of the CRISPR interference (CRISPRi) system as a strategy to inhibit bacterial biofilm [242,243]. Using the CRISPRi method, Zuberi et al. demonstrated that inhibition of genes that are directly involved in quorum sensing could lead to the inhibition of biofilm formation in *E. coli* [242]. Taking a similar step using *P. fluorescens* as a model, Noirot-Gros et al. (2019) investigated the gene network that leads to the disruption in the biofilm formation [243]. It seems that the silencing of bacterial genes responsible in the formation of biofilm by CRISPRi may hold a strategic advantage in the management of biofilm-associated polymicrobial diseases.

## 6. Conclusions and Future Perspectives

Polymicrobial diseases, caused by a complex, complicated, mixed, dual, synergistic, or concurrent milieu of bacteria, fungi, archaea, and viruses, are due to polymicrobial biofilm formation. Polymicrobial infections are resistant to antibacterial agents and usually present delivery barriers at the infection sites, leading to the unsuccessful use of antibiotics. Therefore, they require complex management to modify the clinical course of the disease and avoid selection problems of antimicrobial therapy. Natural products such as EOs, alkaloids, terpenes, quinone, and tannins offer potential in combating polymicrobial diseases. 

Based on numerous published articles discussed in this review, the use of emerging drug delivery systems, such as polymeric NPs, metal NPs, liposomes, SLNs, and others in combination with the knowledge of pathogenesis of infectious diseases, has enabled significant improvement in antimicrobial drug delivery. Furthermore, these drug-delivery systems have been considered as promising approaches to treat several infectious polymicrobial diseases. These delivery approaches have shown outstanding results in the treatment of diseases caused by bacterial pathogens by allowing responsive, targeted, and combination use of antimicrobial agents. It is projected that these delivery approaches will continue producing developments to delivery systems of antimicrobial agents for efficient, cost-effective therapeutics, patient compliance, and selective delivery in the treatment of various infectious diseases. However, further studies including clinical trials of these approaches are required to investigate their effectiveness. Moreover, strategies to modify colonization of specific microbes by means of bacteriophage therapy, CRISPR-mediated technologies, and other novel strategies might offer a potential avenue for the successful management of polymicrobial diseases.

## Figures and Tables

**Table 1:** Types of interaction and examples of combination of microorganisms involved in polymicrobial diseases (modified and updated from [7]).

Type of Interaction	Examples of Microorganism Involved		Diseases/Outcomes	Refs.
Synergism	Virus–Virus	Human metapneumovirus with coronavirus or respiratory syncytial virus	SARS, Bronchiolitis	[11,12,13]
Synergism	Virus–Virus	Epstein-Barr virus and retrovirus	Multiple sclerosis	[45]
Synergism	Virus–Virus	HTLV-I, HTLV-II, and/or HIV-1, HIV-2	Respiratory and urinary infections	[46]
Synergism	Virus–Virus	HTLV-I and HTLV-II	AIDS	[25]
Synergism	Virus–Virus	HBV or HCV and HIV-1	Concurrent infection of AIDS with hepatitis	[47]
Synergism	Virus–Bacteria	Herpesvirus and *Pseudomonas aeruginosa*	Kaposi’s Sarcoma	[48]
Synergism	Virus–Bacteria	HIV and *Mycobacterium tuberculosis*	Concurrent infection of AIDS with tuberculosis	[19]
Synergism	Virus–Bacteria	Measles and *M. tuberculosis* and *Staphylococcus aureus*	Measles	[17]
Synergism	Bacteria–Bacteria	*Streptococcus gordonii*, *Fusobacterium nucleatum* and *Porphyromonas gingivalis*	Periodontitis	[49]
Synergism	Bacteria–Fungi	*Stenotrophomonas maltophilia and Aspergillus fumigatus*	Corneal infection	[50]
Synergism	Bacteria–Fungi	*Candida albicans and S. aureus*	Cystic fibrosis, ventilator-associated pneumonia	[51]
Synergism	Fungi–Fungi	*C. albicans* with *C. glabrata*	Denture stomatitis	[52]
Interference	Virus–Virus	Flavivirus and HIV	AIDS	[15]
Interference	Bacteria–Bacteria	*Streptococcus pneumoniae* and *S. aureus*	*Staphylococcus aureus*-related diseases	[53]
Interference	Bacteria–Bacteria	*Streptococcus sanguinis* and *S. gordonii* with *Streptococcus mutans*	Dental caries	[54]
Interference	Bacteria–Fungi	*Porphyromonas* spp. with *C. albicans*	Periodontal diseases	[55]
Interference	Bacteria–Fungi	*P. aeruginosa* with *C. albicans*	Periodontal diseases	[56]
Predisposing infections	Virus–Bacteria	Influenza A virus with *S. pneumoniae* and *S. aureus*	Otitis media	[57,58]
Predisposing infections	Virus–Bacteria	Varicella-zoster virus and *Streptococcus pyogenes*	Invasive group A streptococcal disease	[59]
Additive	Bacteria–Bacteria	*P. aeruginosa*, *S. maltophilia*, *Prevotella oris*, *Fusobacterium gonidoformans*, *Bacteroides fragilis*, *Leptotrichia*-like spp., *Abiotrophia defecta*, *Citrobacter murliniae*, *Lautropia mirabilis*, and *Sarcina ventriculi*	Cystic fibrosis	[60]
Additive	Bacteria–Fungi	Aerobic and anaerobic Gram-positive and Gram-negative bacteria and *Candida* spp.	Periodontal diseases	[1]

HLTV: Human T-cell lymphotropic virus; HBV: Hepatitis B virus; HCV: Hepatitis C virus; HIV: Human immunodeficiency virus; AIDS: Acquired Immunodeficiency Syndrome; SARS: Severe Acute Respiratory Syndrome.

**Table 2:** Classification of antimicrobial agents on the basis of function and site of action.

Types		Examples	Targets	Functions	Recommended Dosage	Common Uses	Refs.
Cell wall synthesis inhibitors		Vancomycin, Beta-lactam drugs, Fosfomycin	Fosfomycin: MurA β-Lactams: Penicillin-binding Proteins (PBPs) Vancomycin: d-Ala-d-Ala terminus	Inhibit cell wall synthesis and promote microbial death	Adult: 1 g every 12 h Children: 40 mg/kg/day	MRSA (Methicillin-Resistant *S. aureus*), Pneumonia, Urinary tract infections, meningitis, Colitis	[61,62,63,64,65,66,67]
Membrane function inhibitors		Polymyxins B and E	Lipopolysaccharide (LPS)	Alter the composition of the cell membrane by demonstrating specialty in the outer surface of Gram-negative bacteria against polysaccharide	Intravenous polymyxin B: 1.5 to 2.5 mg/kg/day CMS (Colistimethate sodium): 6.67—13.3 mg/kg/day	Intestinal infections by multidrug-resistant Gram-negative bacteria	[34,35,68,69,70,71]
Protein synthesis inhibitors		Tetracyclines, macrolides, Aminoglycoside antibiotics	Aminoglycoside: 30S ribosome Macrolides: 50S ribosome Tetracyclines: 30S ribosome	1. Ribosomal structure is altered by aminoglycosides. 2. Macrolides impede peptidyl transfer 3. Protein translation is disturbed by tetracyclines	Adults: 1 g/day Pediatric patients: 25–50 mg/kg In severe infections: 2 g/day	Pelvic inflammatory disease, Lyme disease, Pneumonia, Cholera	[36,40,72]
Nucleic acid synthesis inhibitors	DNA synthesis inhibitor	Metronidazole, Quinolones, Nitrofurantoin	Quinolones: DNA gyrase, metronidazole: DNA strands	The synthetic quinolone of antimicrobials is used for the alteration of cell division, DNA synthesis, and mRNA transcription	Recommended daily dose is generally 500 mg	Infections in the urinary tract, Respiratory tract, skin, gastrointestinal (GI) tract, and Bone, and Pyelonephritis	[43,73,74,75,76,77,78]
	RNA synthesis inhibitor	Rifampin	DNA-dependent RNA polymerase	RNA inhibitors interact with the transcription mechanism of the bacteria and build a wall inhibiting RNA elongation	Adults: 10 mg/kg For 10-years-olds: 450 mg once a month	Tuberculosis, Traveler’s diarrhea	[79,80,81,82]
Inhibitors of metabolic pathways		Trimethoprim, Sulfonamide and Dapsone	Trimethoprim: Dihydrofolate reductase Sulfonamide: p-aminobenzoic acid	(1) Decreased affinity of dihydrofolate reductase (2) Intrinsic resistance if use exogenous thymidine (3) Preventing synthesis of folic acid	Trimethoprim and Sulfonamide: 30 mg/kg	Bacterial pneumonia, Prostatic infections, Bacterial meningitis, Traveler’s diarrhea	[83,84,85,86,87]

**Table 3:** Antimicrobial potential of natural products based on experimental studies.

Natural Products	Sources of Natural Products	Target Microbiota	Inhibitory Concentrations (V/V)/Amounts	Outcomes	Refs.
Essential oils (Clove oil)	*Syzygium aromaticum* L.	*Streptococcus suis*	0.0125 to 0.2%	MIC50 at 0.5%, MIC90 at 0.1%	[153]
	*Eugenia caryophyllata*	*Aspergillus niger*, *Fusarium oxysporum*	350 to 450 ppm	Reduced the growth of *A. niger* from 50% to 70%, and *F. oxysporum* to 40%	[154]
	n. m.	*E. coli*	n. m.	Clove oil did not show any activity against *E. coli*, having a negative effect on the same.	[155]
	*S. aromaticum* L.	*Vibrio harveyi* (FP8370), *Edwardsiella tarda* (ED47)	0.125 to 0.5%	Clove oil inhibited the growth of Gram negative pathogenic bacteria	[156]
	*S. aromaticum* L.	*Streptococcus iniae* (S186), *Lactococcus garvieae* (FP5245)	0.25 to 0.5%	Clove oil inhibited the growth of Gram positive pathogenic bacteria	[156]
	n. m.	*Trichophyton mentagrophytes*, *Microsporum canis*, *Aspergillus flavus* and *C. albicans*	10 to 100%	Showed strong antifungal activity against tested isolated fungi	[157]
Alkaloids	*Callistemon citrinus*	*S. aureus*, *P. aeruginosa*	0.0025 and 0.21 mg/mL	*C. citrinus*-derived alkaloid extracts at 0.0025 and 0.21 mg/mL showed MIC on *S. aureus* and *P. aeruginosa*, respectively	[104]
	*Vernonia adoensis*	*S. aureus*, *P. aeruginosa*	0.21 and 0.42 mg/mL	*V. adoensis* derived alkaloid extracts at 0.21 and 0.42 mg/mL showed MIC on *S. aureus* and *P. aeruginosa,* respectively	[104]
Terpenes	*Helichrysum italicum*	Resistant *Enterobacter* *aerogenes*	2.5%	Efflux pump inhibition	[158]
	*Polyalthia longifolia*	MRSA	2.5 to 10 µg/mL	Antibiotic potentiation efflux pump modulation	[159]
	*Callicarpa farinosa*	MRSA	2 to 512 μg/mL	Growth inhibition detected	[160]
Phenolic compounds	*Guazuma ulmifolia* Lam.	*Trypanosoma cruzi*, *L. brasiliensis*, *L. infantum*	500 µg/mL	Displayed higher leishmanicidal activity due to the presence of quercetin	[122]
	n. m.	*S. aureus*	1750 μg/mL	Antimicrobial activity detected against four pathogenic bacteria	[161]
		*P. aeruginosa*	500 mg/mL		
		*Listeria monocytogenes*	2000 mg/mL		
		*E. coli*	1500 mg/mL		
Quinones	*Nigella sativa*	*P. aeruginosa*, *S. aureus*, *Bacillus subtilis*, *E. coli*	1.56 to 100 μg/mL	Inhibited biofilm formation	[132]
Flavonoids	*Embelia ribes*	*Bacillus cereus*, *Micrococcus luteus*, *S. aureus*, *E. coli*	1.9 ± 0.1 g	Embelin offered a remarkable bacteriostatic and bactericidal activity	[134]
	n. m.	*Enterococcus faecalis*, *S. aureus*, *P. aeruginosa*, *E. coli*	500–1000 µg/mL	Quercetin and rutin demonstrated antibacterial activity against all bacterial strains	[162]

MIC: Minimum inhibitory concentration; MRSA: Methicillin-resistant *Staphylococcus aureus*; n. m.: Not mentioned.

## Data Availability

Available data are presented in the manuscript.

## References

[1] Peters B.M., Jabra-Rizk M.A., O’May G.A., Costerton J.W., Shirtliff M.E. (2012). Polymicrobial interactions: Impact on pathogenesis and human disease. Clin. Microbiol. Rev..

[2] Lu X., Kurago Z., Brogden K.A. (2006). Effects of polymicrobial communities on host immunity and response. FEMS Microbiol. Lett..

[3] Manson J.M., Rauch M., Gilmore M.S., Huffnagle G.B., Noverr M.C. (2008). The Commensal Microbiology of the Gastrointestinal Tract. GI Microbiota and Regulation of the Immune System.

[4] Aas J.A., Paster B.J., Stokes L.N., Olsen I., Dewhirst F.E. (2005). Defining the normal bacterial flora of the oral cavity. J. Clin. Microbiol..

[5] Peleg A.Y., Hogan D.A., Mylonakis E. (2010). Medically important bacterial-fungal interactions. Nat. Rev. Microbiol..

[6] Krüger W., Vielreicher S., Kapitan M., Jacobsen I.D., Niemiec M.J. (2019). Fungal-Bacterial Interactions in Health and Disease. Pathogens.

[7] Brogden K.A., Guthmiller J.M., Taylor C.E. (2005). Human polymicrobial infections. Lancet.

[8] Eyler R.F., Shvets K. (2019). Clinical Pharmacology of Antibiotics. Clin. J. Am. Soc. Nephrol..

[9] Spellberg B., Bartlett J.G., Gilbert D.N. (2013). The Future of Antibiotics and Resistance. N. Engl. J. Med..

[10] Mohr K.I. (2016). History of Antibiotics Research. Curr. Top. Microbiol. Immunol..

[11] Greensill J., McNamara P.S., Dove W., Flanagan B., Smyth R.L., Hart C.A. (2003). Human Metapneumovirus in Severe Respiratory Syncytial Virus Bronchiolitis. Emerg. Infect. Dis. J..

[12] Chan P.K., Tam J.S., Lam C.W., Chan E., Wu A., Li C.K., Buckley T.A., Ng K.C., Joynt G.M., Cheng F.W. (2003). Human metapneumovirus detection in patients with severe acute respiratory syndrome. Emerg. Infect. Dis..

[13] Cuevas L.E., Nasser A.M.B., Dove W., Gurgel R.Q., Greensill J., Hart C.A. (2003). Human metapneumovirus and respiratory syncytial virus, Brazil. Emerg. Infect. Dis..

[14] Nainu F., Trenerry A., Johnson K.N. (2019). Wolbachia-mediated antiviral protection is cell-autonomous. J. Gen. Virol..

[15] Williams C.F., Klinzman D., Yamashita T.E., Xiang J., Polgreen P.M., Rinaldo C., Liu C., Phair J., Margolick J.B., Zdunek D. (2004). Persistent GB Virus C Infection and Survival in HIV-Infected Men. N. Engl. J. Med..

[16] Schneider-Schaulies S., Niewiesk S., Schneider-Schaulies J., ter Meulen V. (2001). Measles virus induced immunosuppression: Targets and effector mechanisms. Curr. Mol. Med..

[17] Slifka M.K., Homann D., Tishon A., Pagarigan R., Oldstone M.B.A. (2003). Measles virus infection results in suppression of both innate and adaptive immune responses to secondary bacterial infection. J. Clin. Investig..

[18] Fujinami R.S., Sun X., Howell J.M., Jenkin J.C., Burns J.B. (1998). Modulation of Immune System Function by Measles Virus Infection: Role of Soluble Factor and Direct Infection. J. Virol..

[19] Lawn S.D. (2004). AIDS in Africa: The impact of coinfections on the pathogenesis of HIV-1 infection. J. Infect..

[20] Bell L.C.K., Noursadeghi M. (2018). Pathogenesis of HIV-1 and Mycobacterium tuberculosis co-infection. Nat. Rev. Microbiol..

[21] Van geertruyden J.P. (2014). Interactions between malaria and human immunodeficiency virus anno 2014. Clin. Microbiol. Infect..

[22] Kwenti T.E. (2018). Malaria and HIV coinfection in sub-Saharan Africa: Prevalence, impact, and treatment strategies. Res. Rep. Trop. Med..

[23] Limper A.H., Adenis A., Le T., Harrison T.S. (2017). Fungal infections in HIV/AIDS. Lancet Infect. Dis..

[24] Bowen L.N., Smith B., Reich D., Quezado M., Nath A. (2016). HIV-associated opportunistic CNS infections: Pathophysiology, diagnosis and treatment. Nat. Rev. Neurol..

[25] Murphy E.L., Wang B., Sacher R.A., Fridey J., Smith J.W., Nass C.C., Newman B., Ownby H.E., Garratty G., Hutching S.T. (2004). Respiratory and urinary tract infections, arthritis, and asthma associated with HTLV-I and HTLV-II infection. Emerg. Infect. Dis..

[26] Lee K.H., Gordon A., Foxman B. (2016). The role of respiratory viruses in the etiology of bacterial pneumonia: An ecological perspective. Evol. Med. Public Health.

[27] Heikkinen T., Chonmaitree T. (2003). Importance of respiratory viruses in acute otitis media. Clin. Microbiol. Rev..

[28] Schilder A.G.M., Chonmaitree T., Cripps A.W., Rosenfeld R.M., Casselbrant M.L., Haggard M.P., Venekamp R.P. (2016). Otitis media. Nat. Rev. Dis. Primers.

[29] Peltola V.T., McCullers J.A. (2004). Respiratory viruses predisposing to bacterial infections: Role of neuraminidase. Pediatr. Infect. Dis. J..

[30] Das B., Patra S. (2017). Antimicrobials: Meeting the Challenges of Antibiotic Resistance Through Nanotechnology. Nanostruc. Antimicrob. Ther. Nanostruc. Ther. Med. Ser..

[31] Bugg T.D.H., Braddick D., Dowson C.G., Roper D.I. (2011). Bacterial cell wall assembly: Still an attractive antibacterial target. Trends Biotechnol..

[32] Bensen D.C., Rodriguez S., Nix J., Cunningham M.L., Tari L.W. (2012). Structure of MurA (UDP-N-acetylglucosamine enolpyruvyl transferase) from Vibrio fischeri in complex with substrate UDP-N-acetylglucosamine and the drug fosfomycin. Acta Crystallogr. Sect. F Struct. Biol. Cryst. Commun..

[33] Silhavy T.J., Kahne D., Walker S. (2010). The bacterial cell envelope. Cold Spring Harb. Perspect. Biol..

[34] Ullah H., Ali S. (2017). Classification of Anti-Bacterial Agents and Their Functions. Antibact. Agents.

[35] Poirel L., Jayol A., Nordmanna P. (2017). Polymyxins: Antibacterial activity, susceptibility testing, and resistance mechanisms encoded by plasmids or chromosomes. Clin. Microbiol. Rev..

[36] Kohanski M.A., Dwyer D.J., Collins J.J. (2010). How antibiotics kill bacteria: From targets to networks. Nat. Rev. Microbiol..

[37] Poehlsgaard J., Douthwaite S. (2005). The bacterial ribosome as a target for antibiotics. Nat. Rev. Microbiol..

[38] Wilson D.N. (2014). Ribosome-targeting antibiotics and mechanisms of bacterial resistance. Nat. Rev. Microbiol..

[39] Kotra L.P., Haddad J., Mobashery S. (2000). Aminoglycosides: Perspectives on mechanisms of action and resistance and strategies to counter resistance. Antimicrob. Agents Chemother..

[40] Chopra I., Roberts M. (2001). Tetracycline antibiotics: Mode of action, applications, molecular biology, and epidemiology of bacterial resistance. Microbiol. Mol. Biol. Rev..

[41] Bhattacharjee M.K. (2016). Antibiotics That Inhibit Nucleic Acid Synthesis. Chemistry of Antibiotics and Related Drugs.

[42] Campbell E.A., Korzheva N., Mustaev A., Murakami K., Nair S., Goldfarb A., Darst S.A. (2001). Structural Mechanism for Rifampicin Inhibition of Bacterial RNA Polymerase. Cell.

[43] Aldred K.J., Kerns R.J., Osheroff N. (2014). Mechanism of quinolone action and resistance. Biochemistry.

[44] Masters P.A., O’Bryan T.A., Zurlo J., Miller D.Q., Joshi N. (2003). Trimethoprim-Sulfamethoxazole Revisited. Arch. Intern. Med..

[45] Haahr S., Munch M. (2000). The association between multiple sclerosis and infection with Epstein-Barr virus and retrovirus. J. Neurovirol..

[46] Lewis M.J., Gautier V.W., Wang X.P., Kaplan M.H., Hall W.W. (2000). Spontaneous production of C-C chemokines by individuals infected with human T lymphotropic virus type II (HTLV-II) alone and HTLV-II/HIV-1 coinfected individuals. J. Immunol..

[47] Allory Y., Charlotte F., Benhamou Y., Opolon P., Le Charpentier Y., Poynard T. (2000). Impact of human immunodeficiency virus infection on the histological features of chronic hepatitis C: A case-control study. The MULTIVIRC group. Hum. Pathol..

[48] Markazi A., Bracci P.M., McGrath M., Gao S.J. (2020). *Pseudomonas aeruginosa* Stimulates Inflammation and Enhances Kaposi’s Sarcoma Herpesvirus-Induced Cell Proliferation and Cellular Transformation through both Lipopolysaccharide and Flagellin. mBio.

[49] El-Awady A., de Sousa Rabelo M., Meghil M.M., Rajendran M., Elashiry M., Stadler A.F., Foz A.M., Susin C., Romito G.A., Arce R.M. (2019). Polymicrobial synergy within oral biofilm promotes invasion of dendritic cells and survival of consortia members. NPJ Biofilms Microbiomes.

[50] Cho B.J., Lee G.J., Ha S.Y., Seo Y.H., Tchah H. (2002). Co-infection of the human cornea with *Stenotrophomonas maltophilia* and *Aspergillus fumigatus*. Cornea.

[51] Todd O.A., Peters B.M. (2019). *Candida albicans* and *Staphylococcus aureus* Pathogenicity and Polymicrobial Interactions: Lessons beyond Koch’s Postulates. J. Fungi.

[52] Costa-Orlandi C.B., Sardi J.C.O., Pitangui N.S., de Oliveira H.C., Scorzoni L., Galeane M.C., Medina-Alarcón K.P., Melo W., Marcelino M.Y., Braz J.D. (2017). Fungal Biofilms and Polymicrobial Diseases. J. Fungi.

[53] Bogaert D., van Belkum A., Sluijter M., Luijendijk A., de Groot R., Rümke H.C., Verbrugh H.A., Hermans P.W. (2004). Colonisation by *Streptococcus pneumoniae* and *Staphylococcus aureus* in healthy children. Lancet.

[54] Becker M.R., Paster B.J., Leys E.J., Moeschberger M.L., Kenyon S.G., Galvin J.L., Boches S.K., Dewhirst F.E., Griffen A.L. (2002). Molecular analysis of bacterial species associated with childhood caries. J. Clin. Microbiol..

[55] Sztukowska M.N., Dutton L.C., Delaney C., Ramsdale M., Ramage G., Jenkinson H.F., Nobbs A.H., Lamont R.J. (2018). Community Development between *Porphyromonas gingivalis* and *Candida albicans* Mediated by InlJ and Als3. mBio.

[56] García-Contreras R., Pérez-Eretza B., Lira-Silva E., Jasso-Chávez R., Coria-Jiménez R., Rangel-Vega A., Maeda T., Wood T.K. (2014). Gallium induces the production of virulence factors in *Pseudomonas aeruginosa*. Pathog. Dis..

[57] Patel J.A., Nair S., Revai K., Grady J., Chonmaitree T. (2009). Nasopharyngeal acute phase cytokines in viral upper respiratory infection: Impact on acute otitis media in children. Pediatr. Infect. Dis. J..

[58] Wadowsky R.M., Mietzner S.M., Skoner D.P., Doyle W.J., Fireman P. (1995). Effect of experimental influenza A virus infection on isolation of *Streptococcus pneumoniae* and other aerobic bacteria from the oropharynges of allergic and nonallergic adult subjects. Infect. Immun..

[59] Laupland K.B., Davies H.D., Low D.E., Schwartz B., Green K., McGeer A. (2000). Invasive group A streptococcal disease in children and association with varicella-zoster virus infection. Ontario Group A Streptococcal Study Group. Pediatrics.

[60] Rogers G.B., Hart C.A., Mason J.R., Hughes M., Walshaw M.J., Bruce K.D. (2003). Bacterial diversity in cases of lung infection in cystic fibrosis patients: 16S ribosomal DNA (rDNA) length heterogeneity PCR and 16S rDNA terminal restriction fragment length polymorphism profiling. J. Clin. Microbiol..

[61] Wang F., Zhou H., Olademehin O.P., Kim S.J., Tao P. (2018). Insights into key interactions between vancomycin and bacterial cell wall structures. ACS Omega.

[62] Castañeda-García A., Blázquez J., Rodríguez-Rojas A. (2013). Molecular mechanisms and clinical impact of acquired and intrinsic fosfomycin resistance. Antibiotics.

[63] Sarkar P., Yarlagadda V., Ghosh C., Haldar J. (2017). A review on cell wall synthesis inhibitors with an emphasis on glycopeptide antibiotics. MedChemComm.

[64] Bruniera F.R., Ferreira F.M., Saviolli L.R.M., Bacci M.R., Feder D., Pedreira M.D.L.G., Peterlini M.A.S., Azzalis L.A., Junqueira V.B.C., Fonseca F.L.A. (2015). The use of vancomycin with its therapeutic and adverse effects: A review. Eur. Rev. Med. Pharmacol. Sci..

[65] Neelanjana Pandey M.C. (2020). Beta lactam antibiotics. StatPearls [Internet].

[66] Lodise T.P., Lomaestro B., Graves J., Drusano G.L. (2008). Larger vancomycin doses (at least four grams per day) are associated with an increased incidence of nephrotoxicity. Antimicrob. Agents Chemother..

[67] Frymoyer A., Hersh A.L., Benet L.Z., Guglielmo B.J. (2009). Current recommended dosing of vancomycin for children with invasive methicillin-resistant staphylococcus aureus infections is inadequate. Pediatr. Infect. Dis. J..

[68] BA N. (1965). Mechanisms of antibiotic action. Annu. Rev. Microbiol..

[69] Velkov T., Roberts K.D., Nation R.L., Thompson P.E., Li J. (2013). Pharmacology of polymyxins: New insights into an ‘old’ class of antibiotics. Future Microbiol..

[70] Gupta S., Govil D., Kakar P., Prakash O., Arora D., Das S., Govil P., Malhotra A. (2009). Colistin and polymyxin B: A re-emergence. Indian J. Crit. Care Med..

[71] Balaji V., Jeremiah S.S., Baliga P.R. (2011). Polymyxins: Antimicrobial susceptibility concerns and therapeutic options. Indian J. Med Microbiol..

[72] Shutter M.H.A. (2020). Tetracycline. StatPearls [Internet].

[73] Gellert M., Mizuuchi K., O’Dea M.H., Nash H.A. (1976). DNA gyrase: An enzyme that introduces superhelical turns into DNA. Proc. Natl. Acad. Sci. USA.

[74] Drlica K., Snyder M. (1978). Superhelical Escherichia coli DNA: Relaxation by coumermycin. J. Mol. Biol..

[75] Espeli O., Marians K.J. (2004). Untangling intracellular DNA topology. Mol. Microbiol..

[76] Denyer S.P., Hodges N.A., Gorman S.P. (2004). Gorman, Sean. Hugo and Russell’s Pharmaceutical Microbiology. Helicobacter.

[77] Ralph E.D. (1978). The bactericidal activity of nitrofurantoin and metronidazole against anaerobic bacteria. J. Antimicrob. Chemother..

[78] Gupta A., Krishna V. (1993). Clinical Ophthalmology: Contemporary Perspectives.

[79] Calvori C., Frontali L., Leoni L., Tecce G. (1965). Effect of rifamycin on protein synthesis [28]. Nature.

[80] Seijger C., Hoefsloot W., Bergsma I., Van Ingen J., Kuijpers S., Te Brake L., Van Crevel R., Aarnoutse R., Boeree M., Magis-Escurra C. (2019). High-dose rifampicin in tuberculosis: Experiences from a Dutch tuberculosis centre. PLoS ONE.

[81] Steffen R., DuPont H.L. (2019). Rifamycin SV-MMX^®^ as the recommended self-treatment for moderate to severe travellers’ diarrhoea: Reply. J. Travel. Med..

[82] World Health Organization (2010). Guidelines for Treatment of Tuberculosis.

[83] Sykes J.E., Papich M.G. (2013). Chapter 8: Antibacterial Drugs. Canine and Feline Infectious Diseases.

[84] Then R.L. (1982). Mechanisms of resistance to trimethoprim, the sulfonamides, and trimethoprim-sulfamethoxazole. Rev. Infect. Dis..

[85] Weese J.S., Blondeau J.M., Boothe D., Breitschwerdt E.B., Guardabassi L., Hillier A., Lloyd D.H., Papich M.G., Rankin S.C., Turnidge J.D. (2011). Antimicrobial use guidelines for treatment of urinary tract disease in dogs and cats: Antimicrobial guidelines working group of the international society for companion animal infectious diseases. Vet. Med. Int..

[86] Harford C.G., Smith M.R., Wood W.B. (1946). Sulfonamide chemotherapy of combined infection with influenza virus and bacteria. J. Exp. Med..

[87] Scheld W.M., Mandell G.L. (1984). Sulfonamides and Meningitis. JAMA J. Am. Med Assoc..

[88] Neuman H., Forsythe P., Uzan A., Avni O., Koren O. (2018). Antibiotics in early life: Dysbiosis and the damage done. FEMS Microbiol. Rev..

[89] Grill M.F., Maganti R.K. (2011). Neurotoxic effects associated with antibiotic use: Management considerations. Br. J. Clin. Pharm..

[90] Hamamoto H., Urai M., Ishii K., Yasukawa J., Paudel A., Murai M., Kaji T., Kuranaga T., Hamase K., Katsu T. (2015). Lysocin E is a new antibiotic that targets menaquinone in the bacterial membrane. Nat. Chem. Biol..

[91] Cowan M.M. (1999). Plant products as antimicrobial agents. Clin. Microbiol. Rev..

[92] Swamy M.K., Akhtar M.S., Sinniah U.R. (2016). Antimicrobial properties of plant essential oils against human pathogens and their mode of action: An updated review. Evid. Based Complement. Altern. Med..

[93] Galvão L.C.D.C., Furletti V.F., Bersan S.M.F., Da Cunha M.G., Ruiz A.L.T.G., Carvalho J.E.D., Sartoratto A., Rehder V.L.G., Figueira G.M., Teixeira Duarte M.C. (2012). Antimicrobial activity of essential oils against Streptococcus mutans and their antiproliferative effects. Evid. Based Complement. Altern. Med..

[94] Davidson P.M., Branen A.L., Sofos J.N. (2005). Naturally occurring compounds. Antimicrob. Foods.

[95] Carson C.F., Mee B.J., Riley T.V. (2002). Mechanism of action of Melaleuca alternifolia (tea tree) oil on Staphylococcus aureus determined by time-kill, lysis, leakage, and salt tolerance assays and electron microscopy. Antimicrob. Agents Chemother..

[96] Hammer K.A., Carson C.F., Rileya T.V. (2012). Effects of Melaleuca alternifolia (tea tree) essential oil and the major monoterpene component terpinen-4-ol on the development of single- and multistep antibiotic resistance and antimicrobial susceptibility. Antimicrob. Agents Chemother..

[97] Xu J.-G., Liu T., Hu Q.-P., Cao X.-M. (2016). Chemical Composition, Antibacterial Properties and Mechanism of Action of Essential Oil from Clove Buds against *Staphylococcus aureus*. Molecules.

[98] Arora D.S., Kaur J. (1999). Antimicrobial activity of spices. Int. J. Antimicrob. Agents..

[99] Ultee A., Smid E.J. (2001). Influence of carvacrol on growth and toxin production by Bacillus cereus. Int. J. Food Microbiol..

[100] Adorjan B., Buchbauer G. (2010). Biological properties of essential oils: An updated review. Flavour Fragr. J..

[101] Sivropoulou A., Nikolaou C., Papanikolaou E., Kokkini S., Lanaras T., Arsenakis M. (1997). Antimicrobial, Cytotoxic, and Antiviral Activities of Salvia fructicosa Essential Oil. J. Agric. Food Chem..

[102] Elizaquível P., Azizkhani M., Aznar R., Sánchez G. (2013). The effect of essential oils on norovirus surrogates. Food Control.

[103] Böhme K., Barros-Velázquez J., Calo-Mata P., Aubourg S.P. (2014). Antibacterial, antiviral and antifungal activity of essential oils: Mechanisms and applications. Antimicrob. Compd. Curr. Strateg. New Altern..

[104] Mabhiza D., Chitemerere T., Mukanganyama S. (2016). Antibacterial Properties of Alkaloid Extracts from Callistemon citrinus and Vernonia adoensis against Staphylococcus aureus and Pseudomonas aeruginosa *Int*. J. Med. Chem..

[105] Anyanwu A.A., Jimam N.S., Omale S., Wannang N.N. (2017). Antiviral activities of Cucumis metuliferus fruits alkaloids on Infectious Bursal Disease Virus (IBDV). J. Phytopharm..

[106] Moradi M.T., Karimi A., Lorigooini Z. (2018). Alkaloids as the natural anti-influenza virus agents: A systematic review. Toxin Rev..

[107] Mahizan N.A., Yang S.-K., Moo C.-L., Song A.A.-L., Chong C.-M., Chong C.-W., Abushelaibi A., Lim S.-H.E., Lai K.-S. (2019). Terpene Derivatives as a Potential Agent against Antimicrobial Resistance (AMR) Pathogens. Molecules.

[108] Nostro A., Roccaro A.S., Bisignano G., Marino A., Cannatelli M.A., Pizzimenti F.C., Cioni P.L., Procopio F., Blanco A.R. (2007). Effects of oregano, carvacrol and thymol on Staphylococcus aureus and Staphylococcus epidermidis biofilms. J. Med. Microbiol..

[109] Jabra-Rizk M.A., Meiller T.F., James C.E., Shirtliff M.E. (2006). Effect of farnesol on Staphylococcus aureus biofilm formation and antimicrobial susceptibility. Antimicrob. Agents Chemother..

[110] Astani A., Schnitzler P. (2014). Antiviral activity of monoterpenes beta-pinene and limonene against herpes simplex virus in vitro. Iran. J. Microbiol..

[111] Del Rio D., Rodriguez-Mateos A., Spencer J.P.E., Tognolini M., Borges G., Crozier A. (2013). Dietary (poly)phenolics in human health: Structures, bioavailability, and evidence of protective effects against chronic diseases. Antioxid. Redox Signal..

[112] Takó M., Kerekes E.B., Zambrano C., Kotogán A., Papp T., Krisch J., Vágvölgyi C. (2020). Plant phenolics and phenolic-enriched extracts as antimicrobial agents against food-contaminating microorganisms. Antioxidants.

[113] Gyawali R., Ibrahim S.A. (2014). Natural products as antimicrobial agents. Food Control.

[114] Maguire van Seventer J., Hamer D.H., Quah S.R. (2017). Foodborne Diseases. International Encyclopedia of Public Health.

[115] Nazzaro F., Fratianni F., Coppola R. (2013). Quorum sensing and phytochemicals. Int. J. Mol. Sci..

[116] Maddox C.E., Laur L.M., Tian L. (2010). Antibacterial activity of phenolic compounds against the phytopathogen Xylella fastidiosa. Curr. Microbiol..

[117] Huang W.Y., Cai Y.Z., Zhang Y. (2010). Natural phenolic compounds from medicinal herbs and dietary plants: Potential use for cancer prevention. Nutr. Cancer.

[118] Ansari M.A., Anurag A., Fatima Z., Hameed S. (2013). Natural Phenolic Compounds: A Potential Antifungal Agent. Microb. Pathog. Strateg. Combat. Them Sci. Technol. Educ..

[119] Sartini S., Djide M.N., Nainu F. (2019). Correlation phenolic concentration to antioxidant and antibacterial activities of several ethanolic extracts from Indonesia. J. Phys. Conf. Ser..

[120] Zhang L., Chang W., Sun B., Groh M., Speicher A., Lou H. (2011). Bisbibenzyls, a new type of antifungal agent, inhibit morphogenesis switch and biofilm formation through upregulation of DPP3 in Candida albicans. PLoS ONE.

[121] Bouddine L. (2012). Comparative study of the antifungal activity of some essential oils and their major phenolic components against *Aspergillus niger* using three different methods. Afr. J. Biotechnol..

[122] Calixto Júnior J.T., de Morais S.M., Gomez C.V., Molas C.C., Rolon M., Boligon A.A., Athayde M.L., de Morais Oliveira C.D., Tintino S.R., Henrique Douglas M.C. (2016). Phenolic composition and antiparasitic activity of plants from the Brazilian Northeast “Cerrado”. Saudi J. Biol. Sci..

[123] Goel S., Parihar P.S., Meshram V. (2020). Plant-derived quinones as a source of antibacterial and anticancer agents. Bioact. Nat. Prod. Drug Discov..

[124] Silva R.L., Demarque D.P., Dusi R.G., Sousa J.P.B., Albernaz L.C., Espindola L.S. (2020). Residual Larvicidal Activity of Quinones against Aedes aegypti. Molecules.

[125] Gali-Muhtasib H., Roessner A., Schneider-Stock R. (2006). Thymoquinone: A promising anti-cancer drug from natural sources. Int. J. Biochem. Cell Biol..

[126] Kokoska L., Havlik J., Valterova I., Sovova H., Sajfrtova M., Jankovska I. (2008). Comparison of chemical composition and antibacterial activity of Nigella sativa seed essential oils obtained by different extraction methods. J. Food Prot..

[127] Chaieb K., Kouidhi B., Jrah H., Mahdouani K., Bakhrouf A. (2011). Antibacterial activity of Thymoquinone, an active principle of Nigella sativa and its potency to prevent bacterial biofilm formation. BMC Complement. Altern. Med..

[128] Ullah R., Rehman A., Zafeer M.F., Rehman L., Khan Y.A., Khan M.A.H., Khan S.N., Khan A.U., Abidi S.M.A. (2017). Anthelmintic Potential of Thymoquinone and Curcumin on *Fasciola gigantica*. PLoS ONE.

[129] Cetinkaya U., Sezer G., Charyyeva A. (2020). Anti-microsporidial effect of thymoquinone on *Encephalitozoon intestinalis* infection in vitro. Asian Pac. J. Trop. Biomed..

[130] Bassem Yousef S., Manal Mohamed Elhassan T., Waleed Syaed K., Siddig Ibrahim A. (2015). Antimicrobial Effects of Thymoquinone on *Entamoeba histolytica* and *Giardia lamblia*. Pharmacogn. J..

[131] Mahmoudvand H., Tavakoli R., Sharififar F., Minaie K., Ezatpour B., Jahanbakhsh S., Sharifi I. (2015). Leishmanicidal and cytotoxic activities of *Nigella sativa* and its active principle, thymoquinone. Pharm. Biol..

[132] Goel S., Mishra P. (2018). Thymoquinone inhibits biofilm formation and has selective antibacterial activity due to ROS generation. Appl. Microbiol. Biotechnol..

[133] Mossa J.S., El-Feraly F.S., Muhammad I. (2004). Antimycobacterial constituents from Juniperus procera, Ferula communis and Plumbago zeylanica and their in vitro synergistic activity with isonicotinic acid hydrazide. Phytother. Res..

[134] De Paiva S.R., Figueiredo M.R., Aragão T.V., Coelho Kaplan M.A. (2003). Antimicrobial Activity in Vitro of Plumbagin Isolated from Plumbago Species. Mem. Inst. Oswaldo Cruz.

[135] Lorsuwannarat N., Piedrafita D., Chantree P., Sansri V., Songkoomkrong S., Bantuchai S., Sangpairot K., Kueakhai P., Changklungmoa N., Chaichanasak P. (2014). The in vitro anthelmintic effects of plumbagin on newly excysted and 4-weeks-old juvenile parasites of *Fasciola gigantica*. Exp. Parasitol..

[136] Sumsakul W., Plengsuriyakarn T., Chaijaroenkul W., Viyanant V., Karbwang J., Na-Bangchang K. (2014). Antimalarial activity of plumbagin in vitro and in animal models. BMC Complement. Altern. Med..

[137] Sheng Z., Ge S., Gao M., Jian R., Chen X., Xu X., Li D., Zhang K., Chen W.-H. (2020). Synthesis and Biological Activity of Embelin and its Derivatives: An Overview. Mini Rev. Med. Chem..

[138] Chitra M., Devi C.S., Sukumar E. (2003). Antibacterial activity of embelin. Fitoterapia.

[139] Feresin G.E., Tapia A., Sortino M., Zacchino S., Arias A.R.D., Inchausti A., Yaluff G., Rodriguez J., Theoduloz C., Schmeda-Hirschmann G. (2003). Bioactive alkyl phenols and embelin from Oxalis erythrorhiza. J. Ethnopharmacol..

[140] Radhakrishnan N., Gnanamani A., Mandal A.B. (2011). A potential antibacterial agent Embelin, a natural benzoquinone extracted from Embelia ribes. Biol. Med..

[141] Kibrnesh B., Daniel B., Kaleab A. (2015). In vivo Antimalarial Evaluation of Embelin and its Semi-Synthetic Aromatic Amine Derivatives. Pharmacogn. J..

[142] Debebe Y., Tefera M., Mekonnen W., Abebe D., Woldekidan S., Abebe A., Belete Y., Menberu T., Belayneh B., Tesfaye B. (2015). Evaluation of anthelmintic potential of the Ethiopian medicinal plant *Embelia schimperi* Vatke in vivo and in vitro against some intestinal parasites. BMC Complement. Altern. Med..

[143] Elliott M., Chithan K. (2017). The impact of plant flavonoids on mammalian biology: Implications for immunity, inflammation and cancer. The Flavonoids Advances in Research Since 1986.

[144] Cushnie T.P.T., Lamb A.J. (2005). Antimicrobial activity of flavonoids. Int. J. Antimicrob. Agents.

[145] Havsteen B. (1983). Flavonoids, a class of natural products of high pharmacological potency. Biochem. Pharmacol..

[146] Xie Y., Yang W., Tang F., Chen X., Ren L. (2014). Antibacterial Activities of Flavonoids: Structure-Activity Relationship and Mechanism. Curr. Med. Chem..

[147] Mori A., Nishino C., Enoki N., Tawata S. (1987). Antibacterial activity and mode of action of plant flavonoids against Proteus vulgaris and Staphylococcus aureus. Phytochemistry.

[148] Stapleton P.D., Shah S., Hamilton-Miller J.M.T., Hara Y., Nagaoka Y., Kumagai A., Uesato S., Taylor P.W. (2004). Anti-Staphylococcus aureus activity and oxacillin resistance modulating capacity of 3-O-acyl-catechins. Int. J. Antimicrob. Agents.

[149] Kono K., Tatara I., Takeda S., Arakawa K., Hara Y. (1994). Antibacterial activity of epigallocatechin gallate against methicillin-resistant Staphylococcus aureus. Kansenshogaku Zasshi. J. Jpn. Assoc. Infect. Dis..

[150] Aboody M.S.A., Mickymaray S. (2020). Anti-fungal efficacy and mechanisms of flavonoids. Antibiotics.

[151] Seo D.J., Jeon S.B., Oh H., Lee B.H., Lee S.Y., Oh S.H., Jung J.Y., Choi C. (2016). Comparison of the antiviral activity of flavonoids against murine norovirus and feline calicivirus. Food Control.

[152] Li B.Q., Fu T., Dongyan Y., Mikovits J.A., Ruscetti F.W., Wang J.M. (2000). Flavonoid baicalin inhibits HIV-1 infection at the level of viral entry. Biochem. Biophys. Res. Commun..

[153] Wongsawan K., Chaisri W., Tangtrongsup S., Mektrirat R. (2020). Bactericidal Effect of Clove Oil against Multidrug-Resistant *Streptococcus suis* Isolated from Human Patients and Slaughtered Pigs. Pathogens.

[154] Muñoz Castellanos L., Amaya Olivas N., Ayala-Soto J., De La O.C.C.M., Zermeño Ortega M., Sandoval Salas F., Hernández-Ochoa L. (2020). In Vitro and In Vivo Antifungal Activity of Clove (*Eugenia caryophyllata*) and Pepper (*Piper nigrum* L.) Essential Oils and Functional Extracts Against Fusarium oxysporum and Aspergillus niger in Tomato (*Solanum lycopersicum* L.). Int. J. Microbiol..

[155] Santa Packyanathan J., Prakasam G. (2017). Antibacterial effect of clove oil against clinical strains of *Escherichia coli*. J. Pharm. Sci. Res..

[156] Pathirana H., Wimalasena S., De Silva B.C.J., Hossain S., Heo G. (2019). Antibacterial activity of clove essential oil and eugenol against fish pathogenic bacteria isolated from cultured olive flounder (*Paralichthys olivaceus*). Slov. Vet. Res..

[157] Eman-Abdeen E., El-Diasty E.M. (2015). Antifungal activity of clove oil on dermatophytes and other fungi. Int. J. Adv. Res..

[158] Lorenzi V., Muselli A., Bernardini A.F., Berti L., Pagès J.-M., Amaral L., Bolla J.-M. (2009). Geraniol restores antibiotic activities against multidrug-resistant isolates from gram-negative species. Antimicrob. Agents Chemother..

[159] Gupta V.K., Tiwari N., Gupta P., Verma S., Pal A., Srivastava S.K., Darokar M.P. (2016). A clerodane diterpene from *Polyalthia longifolia* as a modifying agent of the resistance of methicillin resistant *Staphylococcus aureus*. Phytomedicine.

[160] Chung P.Y., Chung L.Y., Navaratnam P. (2014). Potential targets by pentacyclic triterpenoids from *Callicarpa farinosa* against methicillin-resistant and sensitive *Staphylococcus aureus*. Fitoterapia.

[161] Borges A., Ferreira C., Saavedra M.J., Simões M. (2013). Antibacterial activity and mode of action of ferulic and gallic acids against pathogenic bacteria. Microb. Drug Resist..

[162] Adamczak A., Ożarowski M., Karpiński T.M. (2020). Antibacterial activity of some flavonoids and organic acids widely distributed in plants. J. Clin. Med..

[163] Fauci A.S., Morens D.M. (2012). The Perpetual Challenge of Infectious Diseases. N. Engl. J. Med..

[164] Farmer P.E. (2013). Chronic Infectious Disease and the Future of Health Care Delivery. N. Engl. J. Med..

[165] Gao W., Thamphiwatana S., Angsantikul P., Zhang L. (2014). Nanoparticle approaches against bacterial infections. Wiley Interdiscip. Rev. Nanomed. Nanobiotechnol..

[166] Baxt L.A. (2014). Bacterial Subversion of Host Innate Immune Pathways. Science.

[167] Modlin R.L., Bloom B.R. (2013). TB or not TB: That is no longer the question. Sci. Transl. Med..

[168] Poulikakos P., Falagas M.E. (2013). Aminoglycoside therapy in infectious diseases. Expert Opin. Pharmacother..

[169] Appelbaum P.C., Hunter P.A. (2000). The fluoroquinolone antibacterials: Past, present and future perspectives. Int. J. Antimicrob. Agents.

[170] Morens D.M., Folkers G.K., Fauci A.S. (2010). The challenge of emerging and re-emerging infectious diseases. Nature.

[171] Zhang R., Hua M., Liu H., Li J. (2021). How to design nanoporous silica nanoparticles in regulating drug delivery: Surface modification and porous control. Mater. Sci. Eng. B Solid-State Mater. Adv. Technol..

[172] Dang Y., Guan J. (2020). Nanoparticle-based drug delivery systems for cancer therapy. Smart Mater. Med..

[173] Miller E.M., Samec T.M., Alexander-Bryant A.A. (2021). Nanoparticle delivery systems to combat drug resistance in ovarian cancer. Nanomed. Nanotechnol. Biol. Med..

[174] Bhattacharya S. (2020). Fabrication of poly(sarcosine), poly (ethylene glycol), and poly (lactic-co-glycolic acid) polymeric nanoparticles for cancer drug delivery. J. Drug Deliv. Sci. Technol..

[175] Figueroa-Espada C.G., Mitchell M.J., Riley R.S., Hofbauer S. (2020). Exploiting the placenta for nanoparticle-mediated drug delivery during pregnancy. Adv. Drug Deliv. Rev..

[176] Petros R.A., Desimone J.M. (2010). Strategies in the design of nanoparticles for therapeutic applications. Nat. Rev. Drug Discov..

[177] Hajji S., Khedir S.B., Hamza-Mnif I., Hamdi M., Jedidi I., Kallel R., Boufi S., Nasri M. (2019). Biomedical potential of chitosan-silver nanoparticles with special reference to antioxidant, antibacterial, hemolytic and in vivo cutaneous wound healing effects. Biochim. Biophys. Acta-Gen. Subj..

[178] Yao Y., Zang Y., Qu J., Tang M., Zhang T. (2019). The toxicity of metallic nanoparticles on liver: The subcellular damages, mechanisms, and outcomes. Int. J. Nanomed..

[179] Rigon R.B., Fachinetti N., Severino P., Santana M.H.A., Chorilli M. (2016). Skin delivery and in vitro biological evaluation of trans-Resveratrol-Loaded solid lipid nanoparticles for skin disorder therapies. Molecules.

[180] Kalhapure R.S., Sikwal D.R., Rambharose S., Mocktar C., Singh S., Bester L., Oh J.K., Renukuntla J., Govender T. (2017). Enhancing targeted antibiotic therapy via pH responsive solid lipid nanoparticles from an acid cleavable lipid. Nanomed. Nanotechnol. Biol. Med..

[181] Dimer F.A., de Souza Carvalho-Wodarz C., Goes A., Cirnski K., Herrmann J., Schmitt V., Pätzold L., Abed N., De Rossi C., Bischoff M. (2020). PLGA nanocapsules improve the delivery of clarithromycin to kill intracellular Staphylococcus aureus and Mycobacterium abscessus. Nanomed. Nanotechnol. Biol. Med..

[182] Boomi P., Ganesan R., Prabu Poorani G., Jegatheeswaran S., Balakumar C., Gurumallesh Prabu H., Anand K., Marimuthu Prabhu N., Jeyakanthan J., Saravanan M. (2020). Phyto-Engineered Gold Nanoparticles (AuNPs) with Potential Antibacterial, Antioxidant, and Wound Healing Activities Under in vitro and in vivo Conditions. Int. J. Nanomed..

[183] Türeli N.G., Torge A., Juntke J., Schwarz B.C., Schneider-Daum N., Türeli A.E., Lehr C.M., Schneider M. (2017). Ciprofloxacin-loaded PLGA nanoparticles against cystic fibrosis P. aeruginosa lung infections. Eur. J. Pharm. Biopharm..

[184] Casciaro B., D’Angelo I., Zhang X., Loffredo M.R., Conte G., Cappiello F., Quaglia F., Di Y.P.P., Ungaro F., Mangoni M.L. (2019). Poly(lactide- co-glycolide) Nanoparticles for Prolonged Therapeutic Efficacy of Esculentin-1a-Derived Antimicrobial Peptides against Pseudomonas aeruginosa Lung Infection: In Vitro and in Vivo Studies. Biomacromolecules.

[185] Choi M., Hasan N., Cao J., Lee J., Hlaing S.P., Yoo J.W. (2020). Chitosan-based nitric oxide-releasing dressing for anti-biofilm and in vivo healing activities in MRSA biofilm-infected wounds. Int. J. Biol. Macromol..

[186] Lee J., Hlaing S.P., Cao J., Hasan N., Yoo J.W. (2020). In vitro and in vivo evaluation of a novel nitric oxide-releasing ointment for the treatment of methicillin-resistant Staphylococcus aureus-infected wounds. J. Pharm. Investig..

[187] dos Santos Ramos M.A., dos Santos K.C., da Silva P.B., de Toledo L.G., Marena G.D., Rodero C.F., de Camargo B.A.F., Fortunato G.C., Bauab T.M., Chorilli M. (2020). Nanotechnological strategies for systemic microbial infections treatment: A review. Int. J. Pharm..

[188] Lee J., Kwak D., Kim H., Kim J., Hlaing S.P., Hasan N., Cao J., Yoo J.W. (2020). Nitric oxide-releasing s-nitrosoglutathione-conjugated poly(Lactic-co-glycolic acid) nanoparticles for the treatment of MRSA-infected cutaneous wounds. Pharmaceutics.

[189] Troiano G., Nolan J., Parsons D., Van Geen Hoven C., Zale S. (2016). A Quality by Design Approach to Developing and Manufacturing Polymeric Nanoparticle Drug Products. AAPS J..

[190] Markwalter C.E., Pagels R.F., Wilson B.K., Ristroph K.D., Prud’homme R.K. (2019). Flash nanoprecipitation for the encapsulation of hydrophobic and hydrophilic compounds in polymeric nanoparticles. J. Vis. Exp..

[191] de Freitas L.M., Calixto G.M.F., Chorilli M., Giusti J.S.M., Bagnato V.S., Soukos N.S., Amiji M.M., Fontana C.R. (2016). Polymeric nanoparticle-based photodynamic therapy for chronic periodontitis in Vivo. Int. J. Mol. Sci..

[192] Esmaeili F., Hosseini-Nasr M., Rad-Malekshahi M., Samadi N., Atyabi F., Dinarvand R. (2007). Preparation and antibacterial activity evaluation of rifampicin-loaded poly lactide-co-glycolide nanoparticles. Nanomed. Nanotechnol. Biol. Med..

[193] Mir M., Ahmed N., Permana A.D., Rodgers A.M., Donnelly R.F., Rehman A.U. (2019). Enhancement in site-specific delivery of carvacrol against methicillin resistant staphylococcus aureus induced skin infections using enzyme responsive nanoparticles: A proof of concept study. Pharmaceutics.

[194] Mir M., Permana A.D., Ahmed N., Khan G.M., Rehman A.u., Donnelly R.F. (2020). Enhancement in site-specific delivery of carvacrol for potential treatment of infected wounds using infection responsive nanoparticles loaded into dissolving microneedles: A proof of concept study. Eur. J. Pharm. Biopharm..

[195] Mir M., Permana A.D., Tekko I.A., McCarthy H.O., Ahmed N., Rehman A.u., Donnelly R.F. (2020). Microneedle liquid injection system assisted delivery of infection responsive nanoparticles: A promising approach for enhanced site-specific delivery of carvacrol against polymicrobial biofilms-infected wounds. Int. J. Pharm..

[196] Permana A.D., Mir M., Utomo E., Donnelly R.F. (2020). Bacterially sensitive nanoparticle-based dissolving microneedles of doxycycline for enhanced treatment of bacterial biofilm skin infection: A proof of concept study. Int. J. Pharm. X.

[197] Said S.S., Aloufy A.K., El-Halfawy O.M., Boraei N.A., El-Khordagui L.K. (2011). Antimicrobial PLGA ultrafine fibers: Interaction with wound bacteria. Eur. J. Pharm. Biopharm..

[198] Chiou S.H., Wu W.T. (2004). Immobilization of Candida rugosa lipase on chitosan with activation of the hydroxyl groups. Biomaterials.

[199] Badran M.M., Alomrani A.H., Harisa G.I., Ashour A.E., Kumar A., Yassin A.E. (2018). Novel docetaxel chitosan-coated PLGA/PCL nanoparticles with magnified cytotoxicity and bioavailability. Biomed. Pharmacother..

[200] Permana A.D., McCrudden M.T.C., Donnelly R.F. (2019). Enhanced intradermal delivery of nanosuspensions of antifilariasis drugs using dissolving microneedles: A proof of concept study. Pharmaceutics.

[201] Permana A.D., Paredes A.J., Volpe-Zanutto F., Anjani Q.K., Utomo E., Donnelly R.F. (2020). Dissolving microneedle-mediated dermal delivery of itraconazole nanocrystals for improved treatment of cutaneous candidiasis. Eur. J. Pharm. Biopharm..

[202] Engin A.B., Hayes A.W. (2018). The impact of immunotoxicity in evaluation of the nanomaterials safety. Toxicol. Res. Appl..

[203] Selvaraj V., Manne N.D.P.K., Arvapalli R., Rice K.M., Nandyala G., Fankenhanel E., Blough E.R. (2015). Effect of cerium oxide nanoparticles on sepsis induced mortality and NF-κB signaling in cultured macrophages. Nanomedicine.

[204] Bing W., Sun H., Wang F., Song Y., Ren J. (2018). Hydrogen-producing hyperthermophilic bacteria synthesized size-controllable fine gold nanoparticles with excellence for eradicating biofilm and antibacterial applications. J. Mater. Chem. B.

[205] Kumar M., Wangoo N., Gondil V.S., Pandey S.K., Lalhall A., Sharma R.K., Chhibber S. (2020). Glycolic acid functionalized silver nanoparticles: A novel approach towards generation of effective antibacterial agent against skin infections. J. Drug Deliv. Sci. Technol..

[206] Permana A.D., Anjani Q.K., Sartini, Utomo E., Volpe-Zanutto F., Paredes A.J., Evary Y.M., Mardikasari S.A., Pratama M.R., Tuany I.N. (2021). Selective delivery of silver nanoparticles for improved treatment of biofilm skin infection using bacteria-responsive microparticles loaded into dissolving microneedles. Mater. Sci. Eng. C.

[207] Torchilin V.P. (2005). Recent advances with liposomes as pharmaceutical carriers. Nat. Rev. Drug Discov..

[208] Rideau E., Dimova R., Schwille P., Wurm F.R., Landfester K. (2018). Liposomes and polymersomes: A comparative review towards cell mimicking. Chem. Soc. Rev..

[209] Meers P., Neville M., Malinin V., Scotto A.W., Sardaryan G., Kurumunda R., Mackinson C., James G., Fisher S., Perkins W.R. (2008). Biofilm penetration, triggered release and in vivo activity of inhaled liposomal amikacin in chronic Pseudomonas aeruginosa lung infections. J. Antimicrob. Chemother..

[210] Sarkar S., Hermes DeSantis E.R., Kuper J. (2007). Resurgence of colistin use. Am. J. Health-Syst. Pharm..

[211] Li Y., Huang L., Tang C., Zhang E., Ding L., Yang L. (2016). Preparation and characterisation of the colistin-entrapped liposome driven by electrostatic interaction for intravenous administration. J. Microencapsul..

[212] Cai W., Liu J., Zheng L., Xu Z., Chen J., Zhong J., Song Z., Xu X., Chen S., Jiao C. (2020). Study on the anti-infection ability of vancomycin cationic liposome combined with polylactide fracture internal fixator. Int. J. Biol. Macromol..

[213] Naguib Y.W., Rodriguez B.L., Li X., Hursting S.D., Williams R.O., Cui Z. (2014). Solid lipid nanoparticle formulations of docetaxel prepared with high melting point triglycerides: In vitro and in vivo evaluation. Mol. Pharm..

[214] Demirel M., Yazan Y., Müller R.H., Kiliç F., Bozan B. (2001). Formulation and in vitro-in vivo evaluation of piribedil solid lipid micro- and nanoparticles. J. Microencapsul..

[215] Costa A., Sarmento B., Seabra V. (2018). Mannose-functionalized solid lipid nanoparticles are effective in targeting alveolar macrophages. Eur. J. Pharm. Sci..

[216] Paranjpe M., Finke J.H., Richter C., Gothsch T., Kwade A., Büttgenbach S., Müller-Goymann C.C. (2014). Physicochemical characterization of sildenafil-loaded solid lipid nanoparticle dispersions (SLN) for pulmonary application. Int. J. Pharm..

[217] Rodenak-Kladniew B., Scioli Montoto S., Sbaraglini M.L., Di Ianni M., Ruiz M.E., Talevi A., Alvarez V.A., Durán N., Castro G.R., Islan G.A. (2019). Hybrid Ofloxacin/eugenol co-loaded solid lipid nanoparticles with enhanced and targetable antimicrobial properties. Int. J. Pharm..

[218] Permana A.D., Tekko I.A., McCrudden M.T.C., Anjani Q.K., Ramadon D., McCarthy H.O., Donnelly R.F. (2019). Solid lipid nanoparticle-based dissolving microneedles: A promising intradermal lymph targeting drug delivery system with potential for enhanced treatment of lymphatic filariasis. J. Control. Release.

[219] Ryan R.P., Fouhy Y., Garcia B.F., Watt S.A., Niehaus K., Yang L., Tolker-Nielsen T., Dow J.M. (2008). Interspecies signalling via the *Stenotrophomonas maltophilia* diffusible signal factor influences biofilm formation and polymyxin tolerance in *Pseudomonas aeruginosa*. Mol. Microbiol..

[220] Harriott M.M., Noverr M.C. (2009). *Candida albicans* and *Staphylococcus aureus* form polymicrobial biofilms: Effects on antimicrobial resistance. Antimicrob. Agents Chemother..

[221] Orazi G., O’Toole G.A. (2017). *Pseudomonas aeruginosa* Alters *Staphylococcus aureus* Sensitivity to Vancomycin in a Biofilm Model of Cystic Fibrosis Infection. mBio.

[222] Hoffman L.R., Déziel E., D’Argenio D.A., Lépine F., Emerson J., McNamara S., Gibson R.L., Ramsey B.W., Miller S.I. (2006). Selection for *Staphylococcus aureus* small-colony variants due to growth in the presence of *Pseudomonas aeruginosa*. Proc. Natl. Acad. Sci. USA.

[223] Adam B., Baillie G.S., Douglas L.J. (2002). Mixed species biofilms of *Candida albicans* and *Staphylococcus epidermidis*. J. Med. Microbiol..

[224] Orazi G., O’Toole G.A. (2019). “It Takes a Village”: Mechanisms Underlying Antimicrobial Recalcitrance of Polymicrobial Biofilms. J. Bacteriol..

[225] Gabrilska R.A., Rumbaugh K.P. (2015). Biofilm models of polymicrobial infection. Future Microbiol..

[226] Verderosa A.D., Totsika M., Fairfull-Smith K.E. (2019). Bacterial Biofilm Eradication Agents: A Current Review. Front. Chem..

[227] De Brucker K., Tan Y., Vints K., De Cremer K., Braem A., Verstraeten N., Michiels J., Vleugels J., Cammue B.P., Thevissen K. (2015). Fungal β-1,3-glucan increases ofloxacin tolerance of *Escherichia coli* in a polymicrobial *E. coli*/*Candida albicans* biofilm. Antimicrob. Agents Chemother..

[228] Harriott M.M., Noverr M.C. (2010). Ability of *Candida albicans* mutants to induce *Staphylococcus aureus* vancomycin resistance during polymicrobial biofilm formation. Antimicrob. Agents Chemother..

[229] Manavathu E.K., Vager D.L., Vazquez J.A. (2014). Development and antimicrobial susceptibility studies of in vitro monomicrobial and polymicrobial biofilm models with *Aspergillus fumigatus* and *Pseudomonas aeruginosa*. BMC Microbiol..

[230] Baltzer S.A., Brown M.H. (2011). Antimicrobial peptides: Promising alternatives to conventional antibiotics. J. Mol. Microbiol. Biotechnol..

[231] Norris E.A. (2010). From Vancomycin to Oritavancin: The Discovery and Development of a Novel Lipoglycopeptide Antibiotic. Anti-Infect. Agents Med. Chem..

[232] Darpo B., Lee S.K., Moon T.E., Sills N., Mason J.W. (2010). Oritavancin, a new lipoglycopeptide antibiotic: Results from a thorough QT study. J. Clin. Pharm..

[233] Wang M., Bhardwaj G., Webster T.J. (2017). Antibacterial properties of PEKK for orthopedic applications. Int. J. Nanomed..

[234] García-Arnáez I., Palla B., Suay J., Romero-Gavilán F., García-Fernández L., Fernández M., Goñi I., Gurruchaga M. (2019). A single coating with antibacterial properties for prevention of medical device-associated infections. Eur. Polym. J..

[235] Kim J.H., Park H., Seo S.W. (2017). In situ synthesis of silver nanoparticles on the surface of PDMS with high antibacterial activity and biosafety toward an implantable medical device. Nano Converg..

[236] Sikder P., Koju N., Ren Y., Goel V.K., Phares T., Lin B., Bhaduri S.B. (2018). Development of single-phase silver-doped antibacterial CDHA coatings on Ti6Al4V with sustained release. Surf. Coat. Technol..

[237] Mc Grath S., von Sinderen D. (2007). Bacteriophage: Genetics and Molecular Biology.

[238] Principi N., Silvestri E., Esposito S. (2019). Advantages and limitations of bacteriophages for the treatment of bacterial infections. Front. Pharmacol..

[239] Sarker S.A., McCallin S., Barretto C., Berger B., Pittet A.C., Sultana S., Krause L., Huq S., Bibiloni R., Bruttin A. (2012). Oral T4-like phage cocktail application to healthy adult volunteers from Bangladesh. Virology.

[240] Cerca N., Oliveira R., Azeredo J. (2007). Susceptibility of *Staphylococcus epidermidis* planktonic cells and biofilms to the lytic action of staphylococcus bacteriophage K. Lett. Appl. Microbiol..

[241] Glonti T., Chanishvili N., Taylor P.W. (2010). Bacteriophage-derived enzyme that depolymerizes the alginic acid capsule associated with cystic fibrosis isolates of *Pseudomonas aeruginosa*. J. Appl. Microbiol..

[242] Zuberi A., Misba L., Khan A.U. (2017). CRISPR Interference (CRISPRi) Inhibition of luxS Gene Expression in *E. coli*: An Approach to Inhibit Biofilm. Front. Cell. Infect. Microbiol..

[243] Noirot-Gros M.-F., Forrester S., Malato G., Larsen P.E., Noirot P. (2019). CRISPR interference to interrogate genes that control biofilm formation in *Pseudomonas fluorescens*. Sci. Rep..

